# Supplementary material for: Distinct regulation of hippocampal neuroplasticity and ciliary genes by corticosteroid receptors
Source: Nat Commun. 2021 Aug 6;12:4737. doi: 10.1038/s41467-021-24967-z (PMC8346558; doi:10.1038/s41467-021-24967-z)
Supplement: Supplementary file 1 — Supplementary Information [file 41467_2021_24967_MOESM1_ESM.pdf]

Distinct regulation of hippocampal neuroplasticity and ciliary genes by corticosteroid receptors

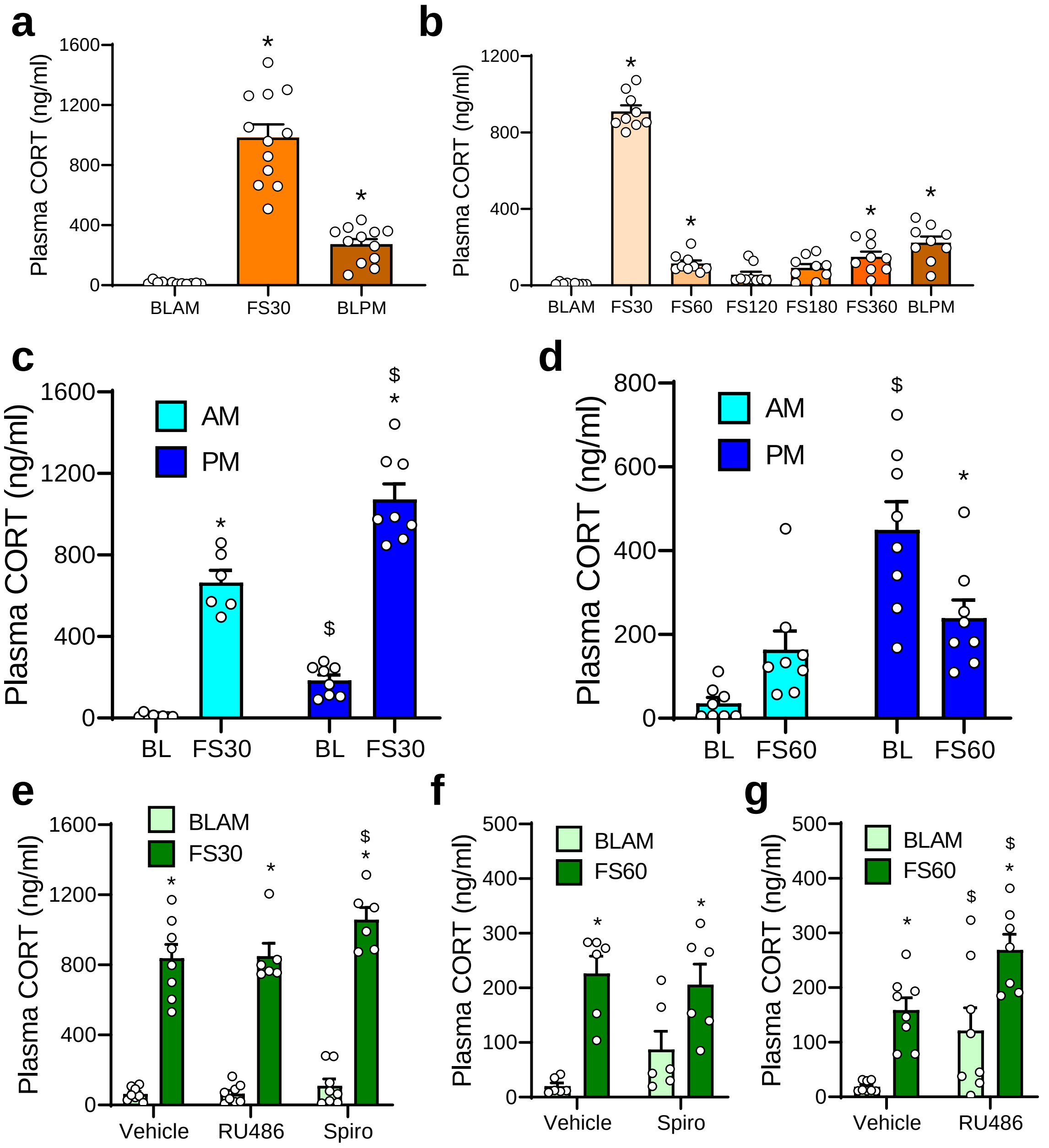

Supplementary Fig. 1: Plasma corticosterone (CORT) levels under baseline conditions and after stress as well as after MR/GR antagonist treatments. Plasma CORT levels (ng/ml; mean ± SEM, n=5-12 rats; dots for number per group) from rats killed under early morning (BLAM) or late afternoon (BLPM) baseline conditions or at different times after the start of FS (15 min, 25 °C water), as indicated in the graphs. Each graph represents the plasma levels in rats killed for the original MR/GR ChIP-Seq (**a**), RNA-Seq (**b**) experiments, validation ChIP-qPCR (**c**) or RNA-qPCR (**d**), or receptor antagonist ChIP-qPCR (**e**) of RNA-qPCR (**f, g**) experiments. Statistical analysis: (a) Oneway ANOVA: F(2,33)=85.07, p<0.0001; (b) Oneway ANOVA: F(6,56)=185.9, P<0.0001; (c) Two-way ANOVA: Effect of stress: F(1,26)=244.0, p<0.0001; Effect of AM/PM: F(1,26)=34.73, P<0.0001; Interaction: F(1,26)=5.658, p=0.0250; (d) Two-way ANOVA: Effect of stress: F(1,28)0.7955, p=0.3800; Effect of AM/PM: F(1,28)=27.62, p<0.0001; Interaction: F(1,28)=13.27, p=0.0011; (e) Two-way ANOVA: Effect of stress: F(1,38)=375.0, p<0.0001; Effect of antagonist: F(2,38)=3.916, p=0.0284; Interaction: F(2,38)=1.659, p=0.2037; (f) Two-way ANOVA: Effect of stress: F(1,20)=29.67, p<0.0001; Effect of antagonist: F(1,20)=0.6154, p=0.4419; Interaction: F(1,20)=2.154, p=0.1578; (g) Two-way ANOVA: Effect of stress: F(1,27)=26.71, p<0.0001; Effect of antagonist: F(1,27)=14.59, p=0.0007; Interaction: F(1,27)=01792, p=0.8945; post-hoc Dunnett's test (a, b) or Bonferroni test (c-g): *, p < 0.05 compared with respective baseline (BLAM/BL) condition; $, p < 0.05 compared with respective AM (c, d) or vehicle (e, f, g) condition. Source data are provided in the Source Data File.

Supplementary Figure 2

Overview of the distribution of MR and GR peaks across all chromosomes for each of the four independent replicates and all physiological conditions studied.

You will find these distributions in the next 24 pages.

Legend:

EXPERIMENTAL GROUP	SAMPLES		INFORMATION RE EXPERIMENTAL GROUP
Baseline AM (BLAM)	A, D, G, P		Rats killed under baseline (non-stress) conditions in the early morning (AM)
FS30	B, E, K, Q		Rats killed 30 min after forced swimming (FS)
Baseline PM (BLPM)	C, F, I, R		Rats killed under baseline conditions in the early evening (PM)

Replicate A-GR-baselineAM:

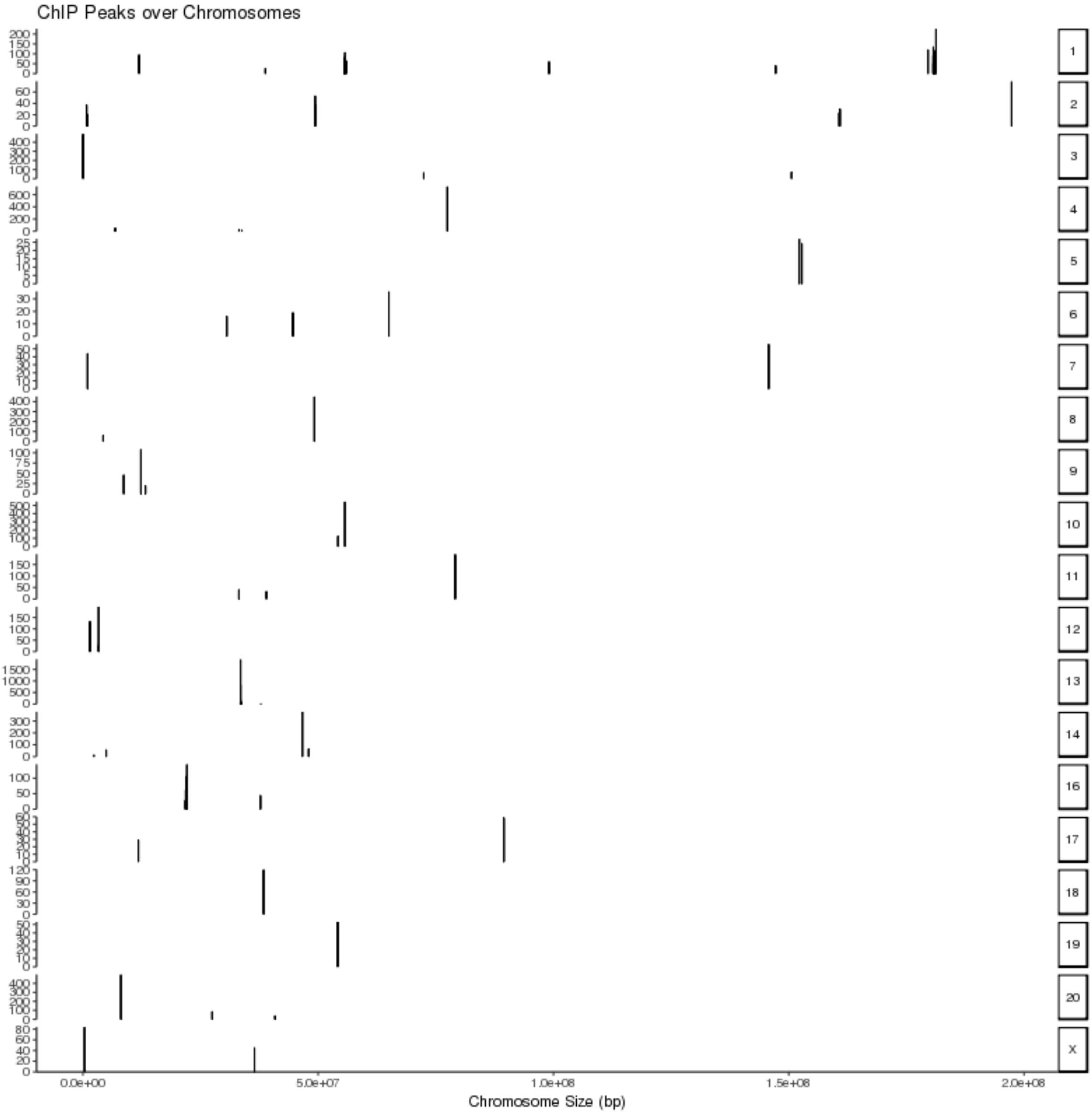

Replicate A-MR-baselineAM:

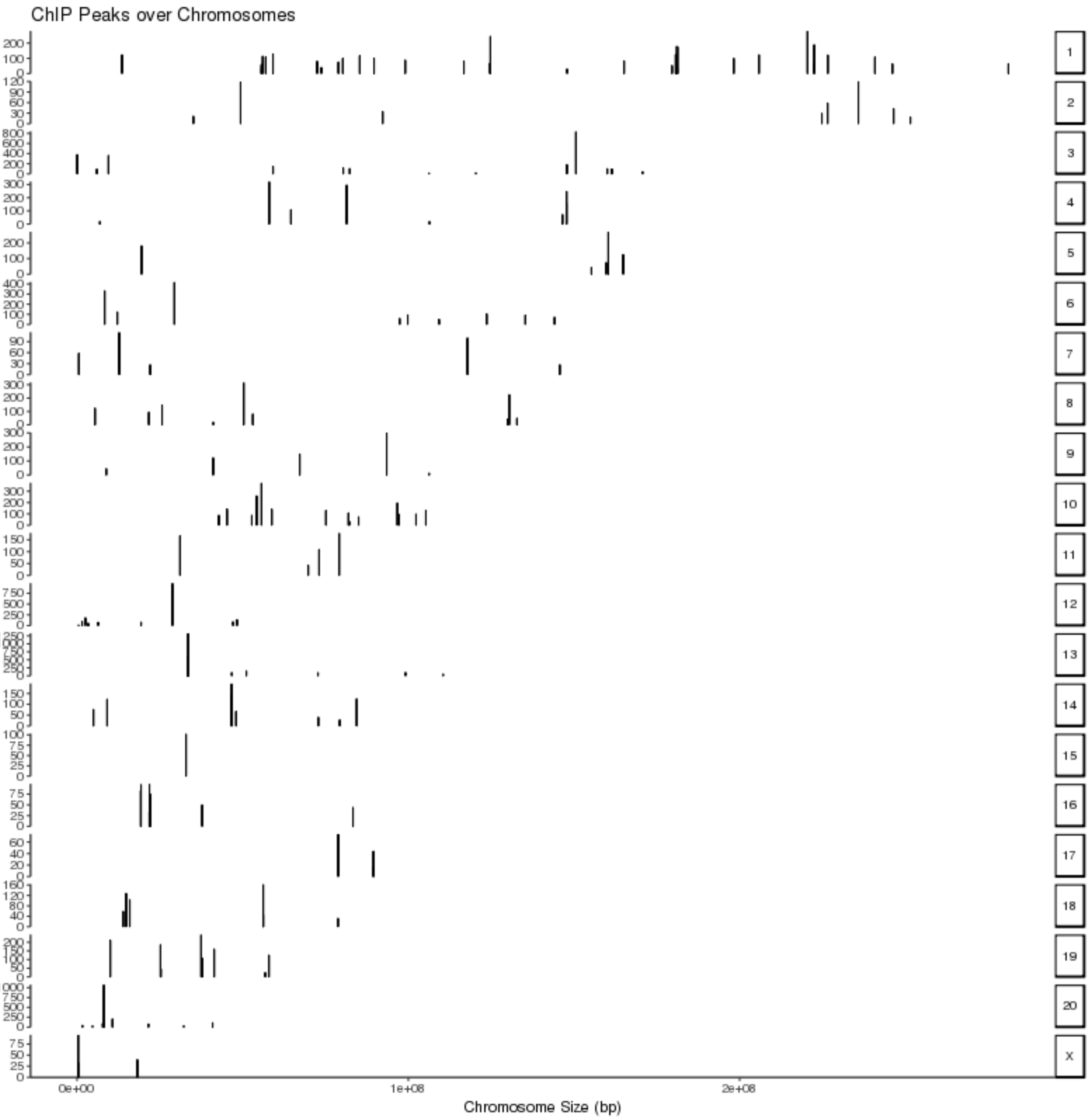

Replicate B-GR-fs30:

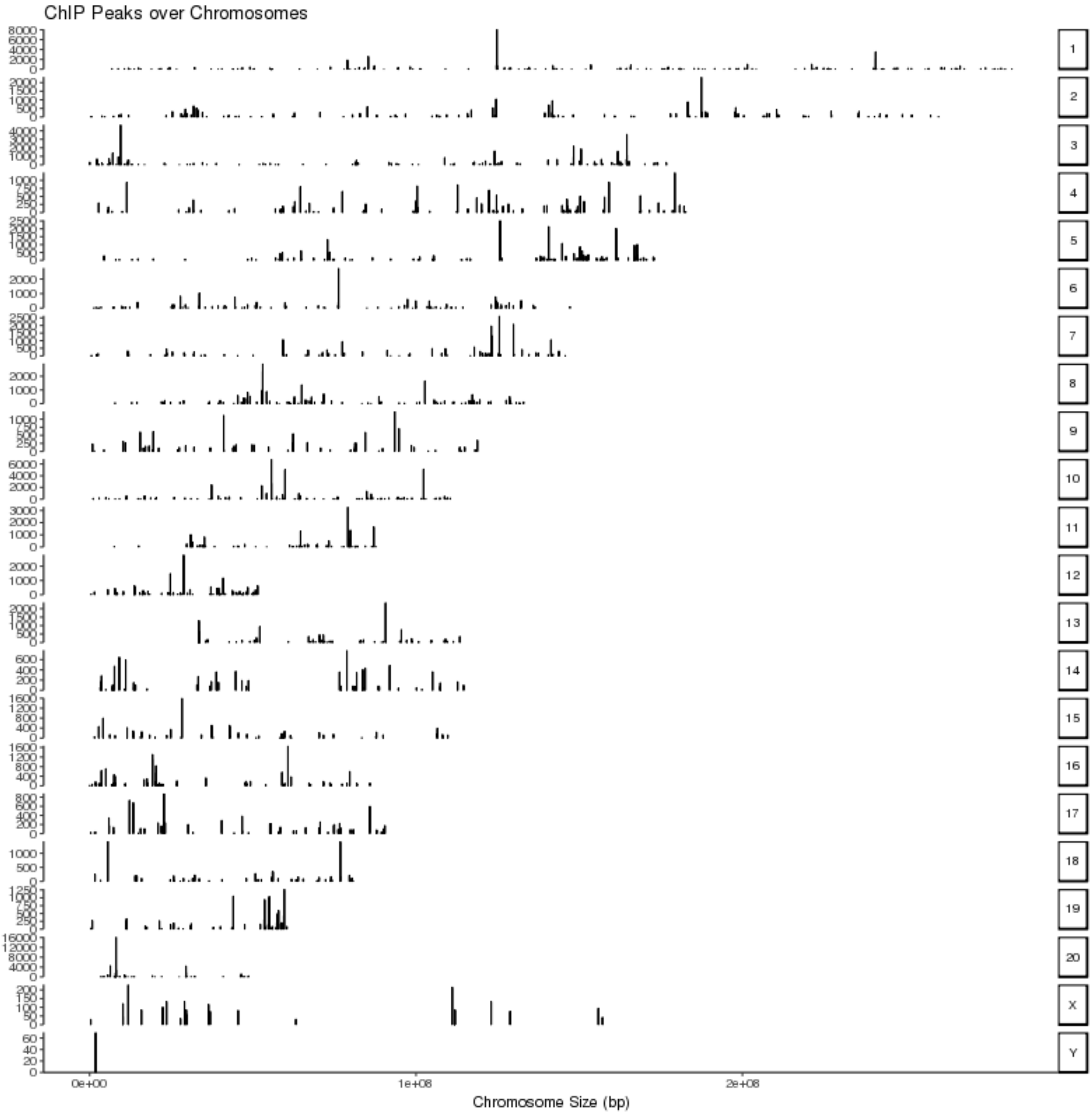

Replicate B-MR-fs30:

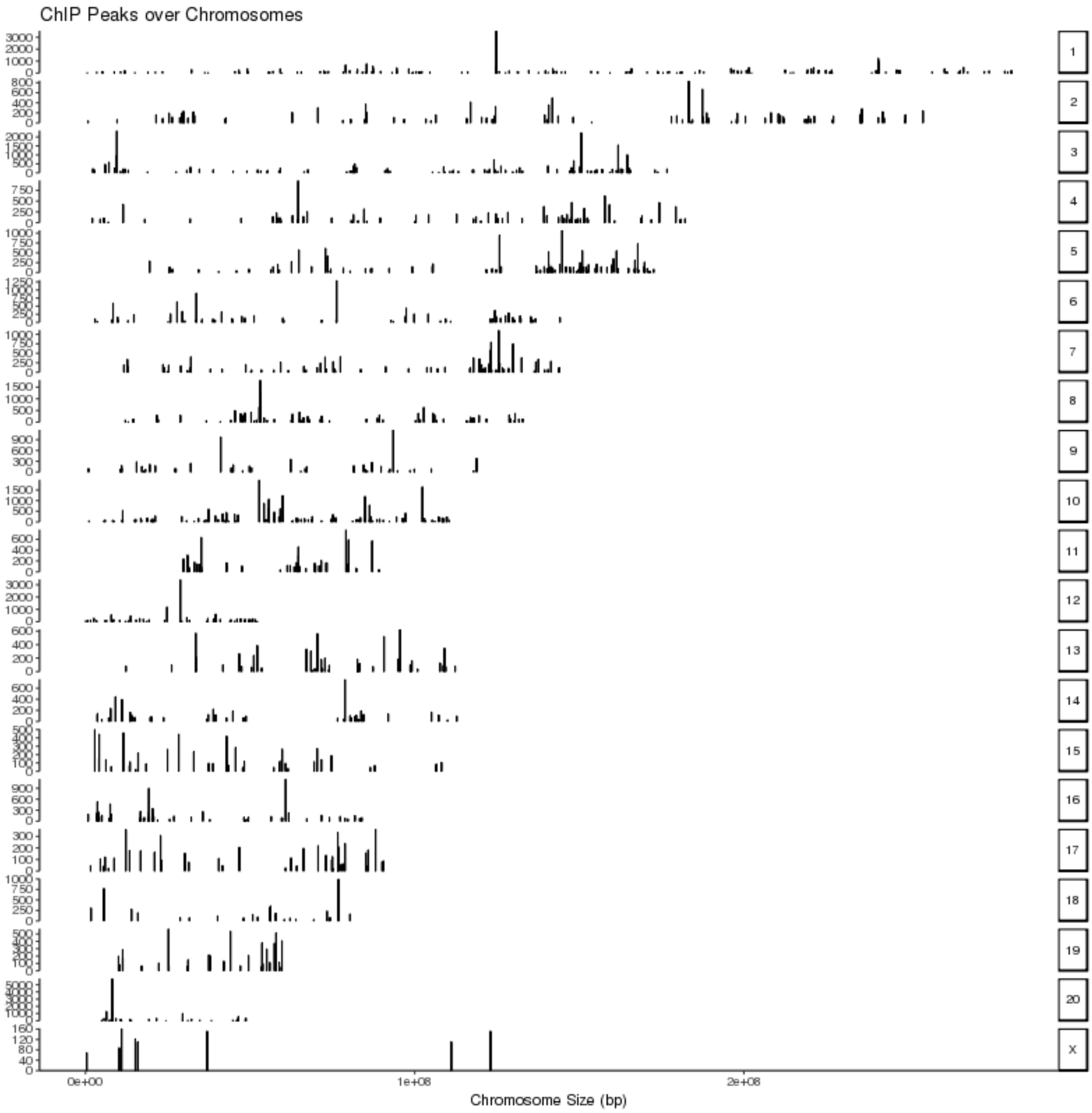

Replicate C-GR-baselinePM:

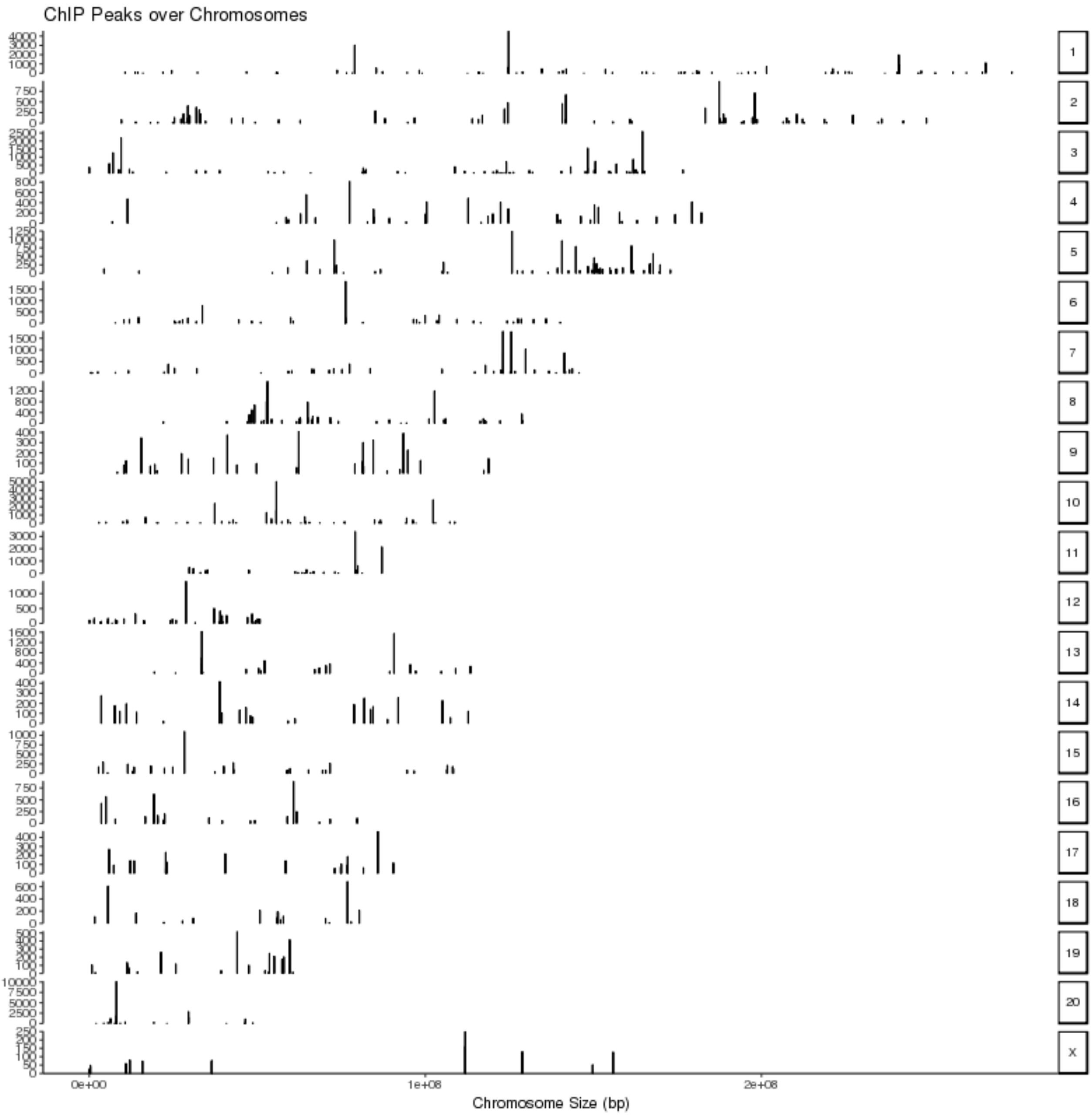

Replicate C-MR-baselinePM:

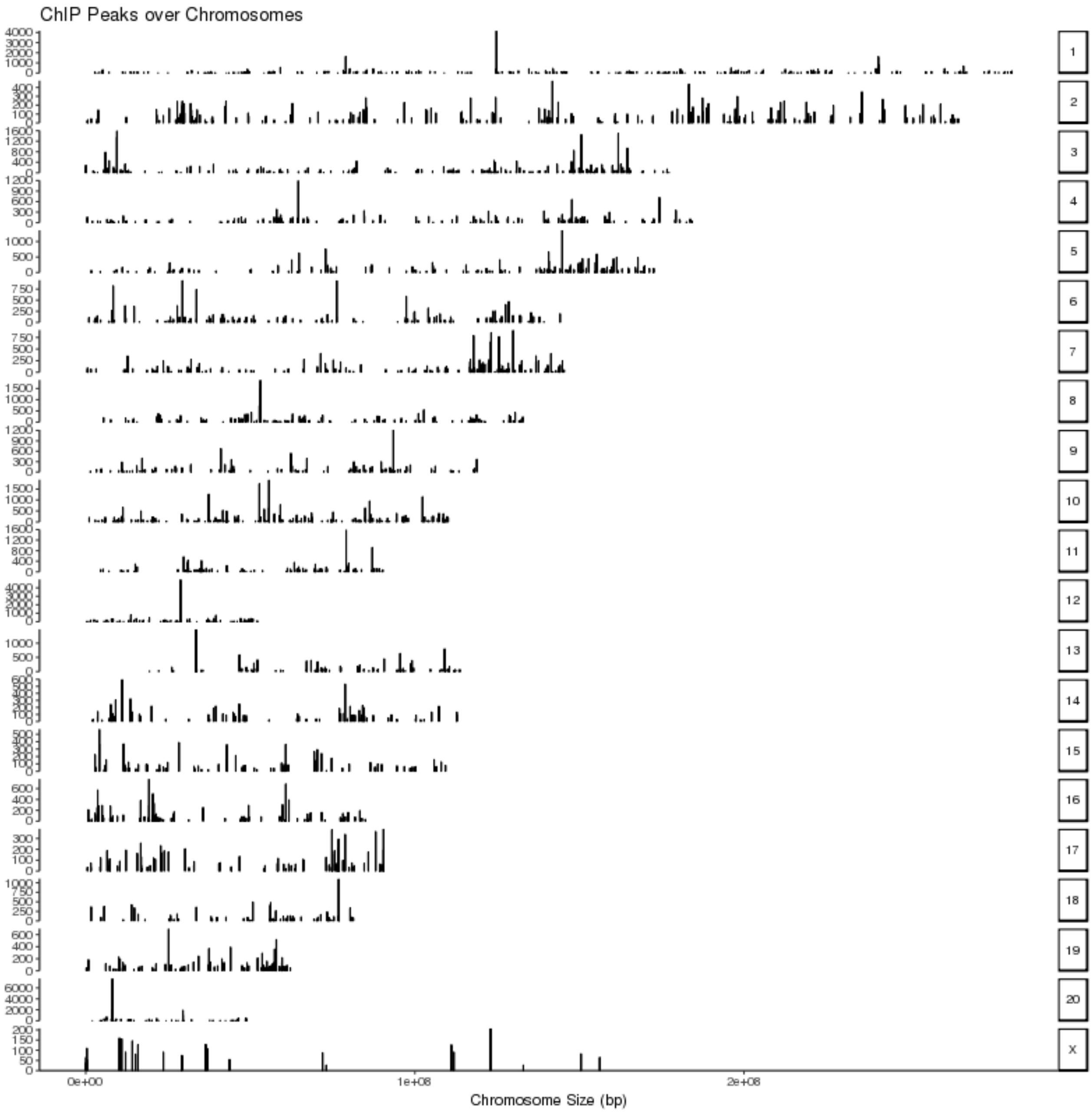

Replicate D-GR-baselineAM:

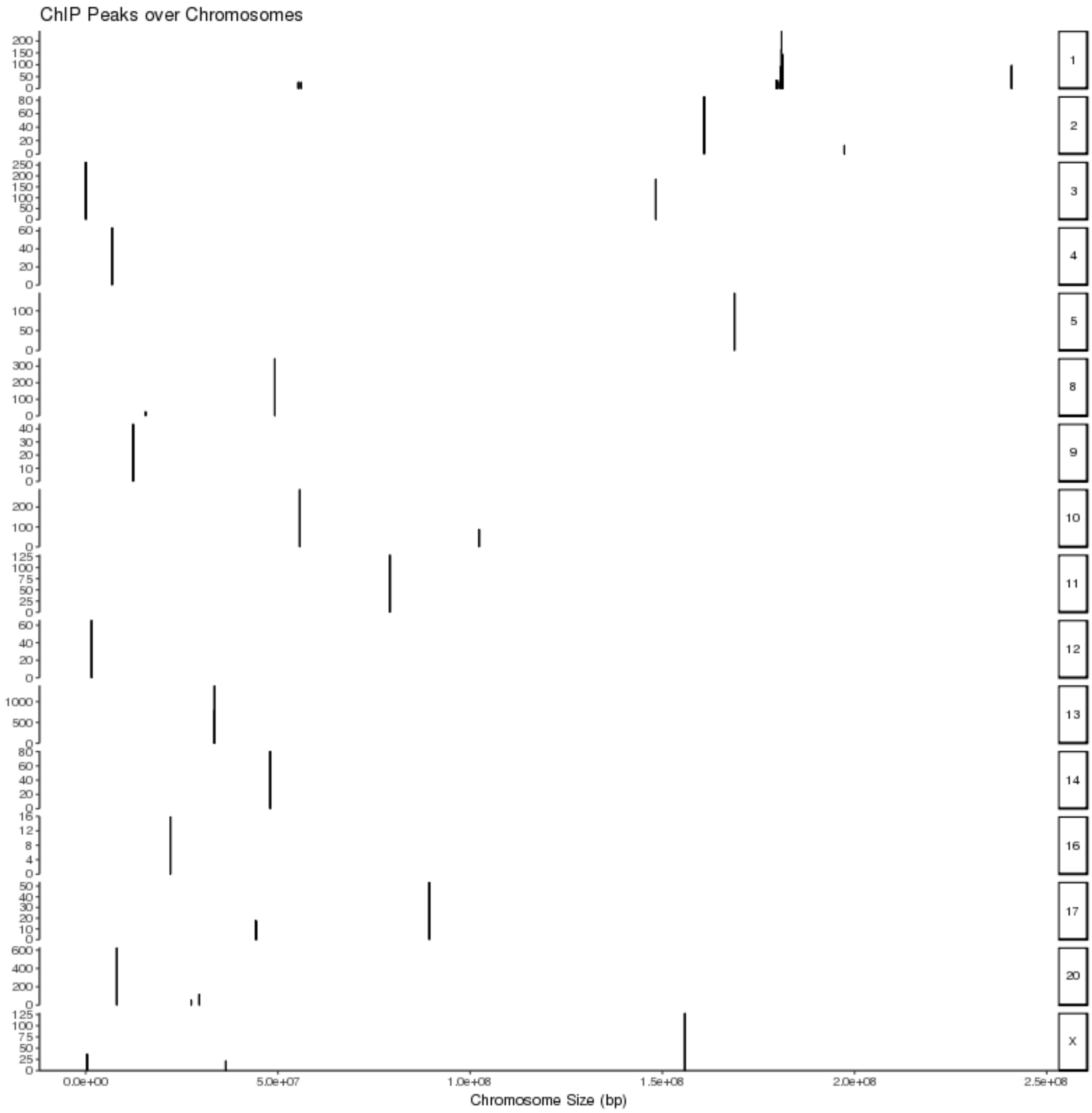

Replicate D-MR-baselineAM:

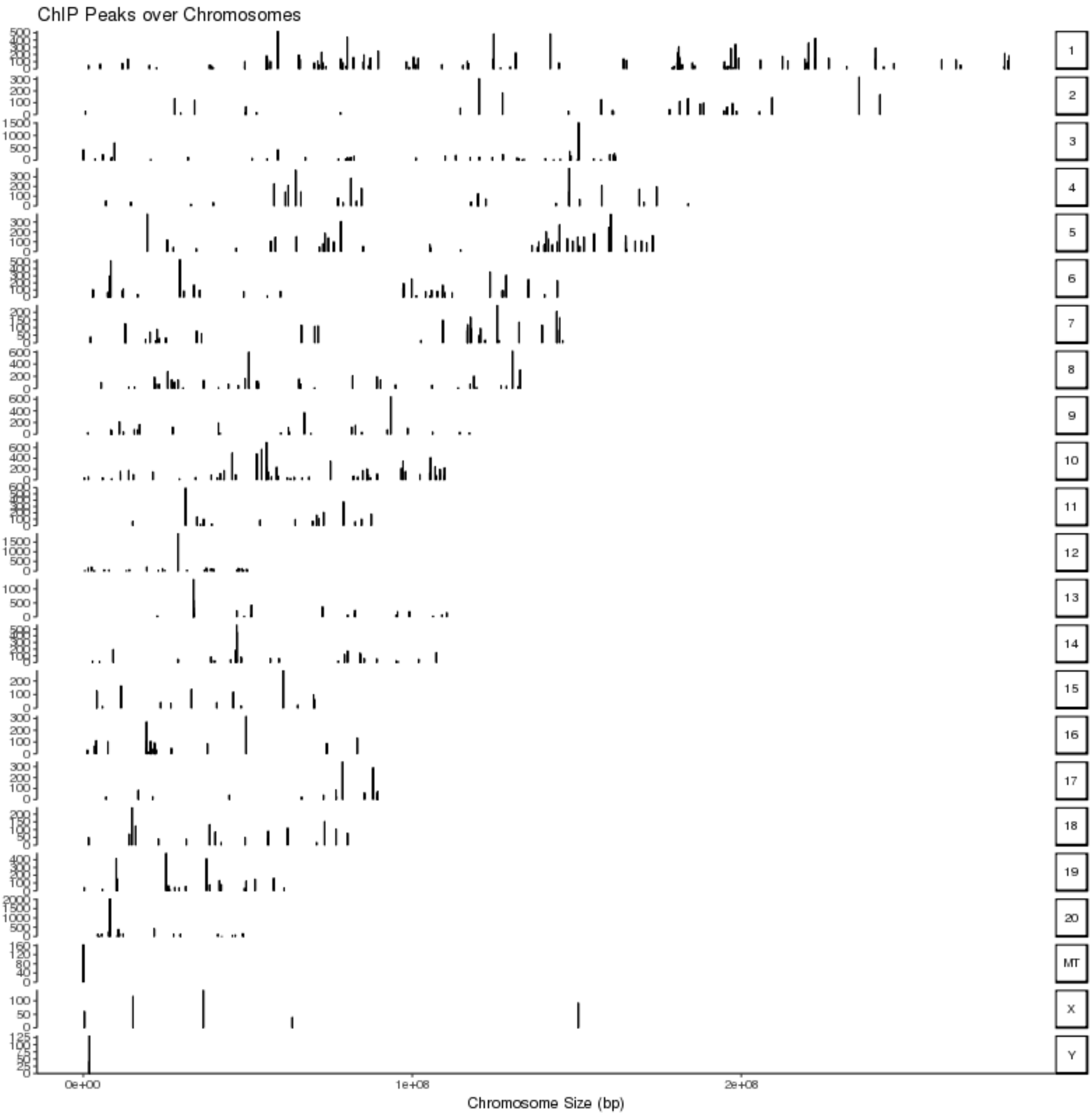

Replicate E-GR-fs30:

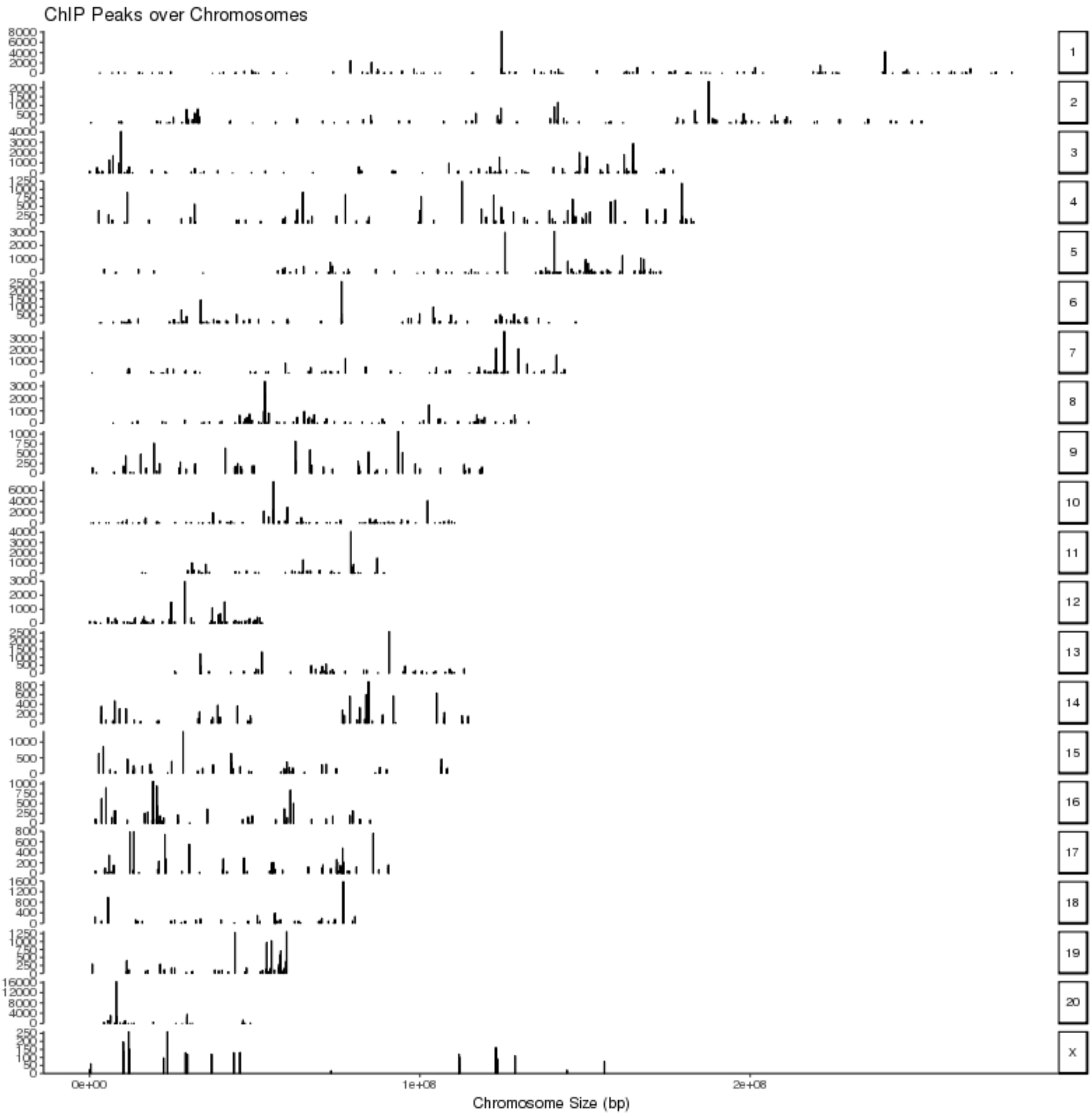

Replicate E-MR-fs30:

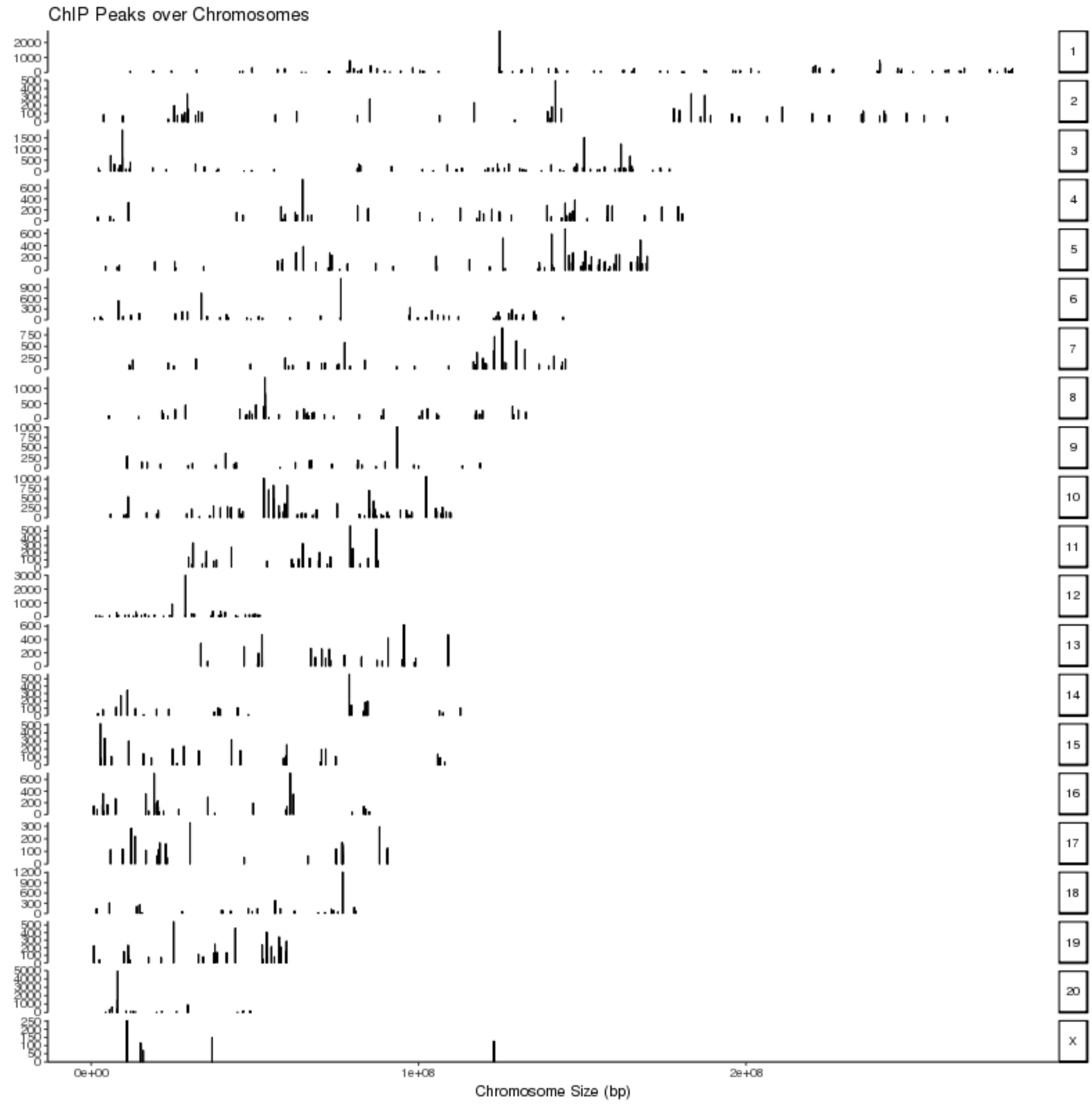

Replicate F-GR-baselinePM:

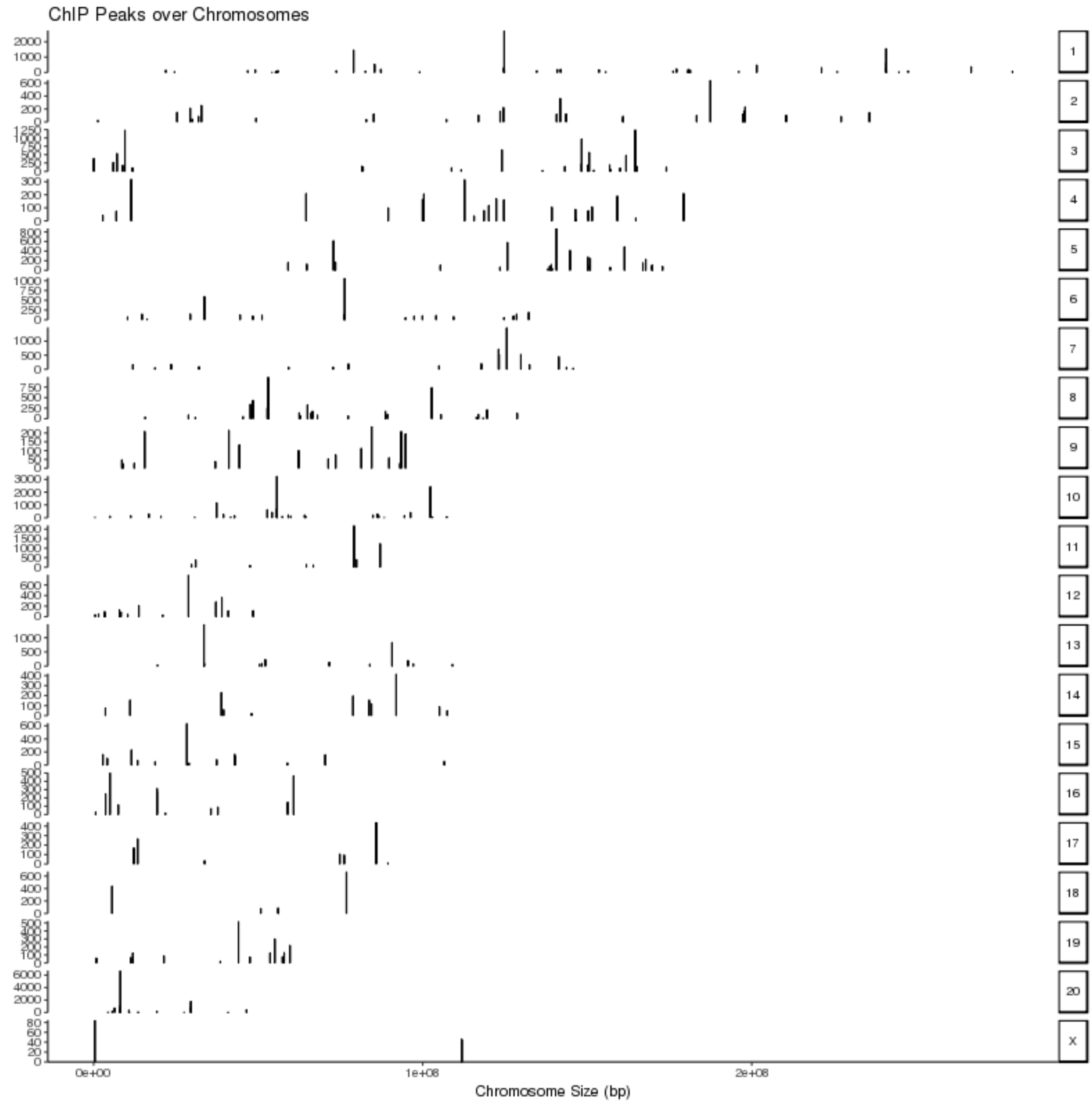

Replicate F-MR-baselinePM:

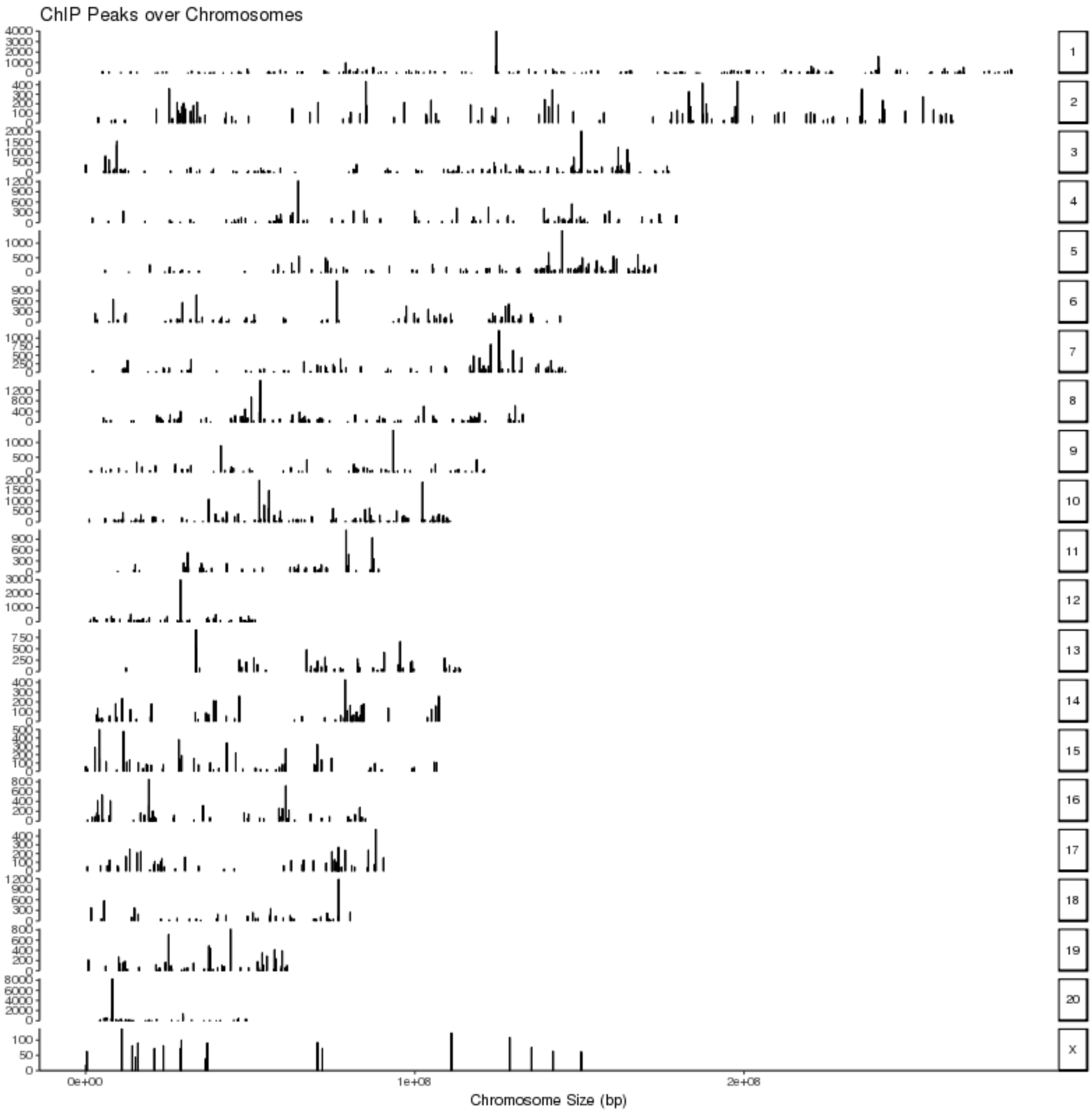

Replicate G-GR-baselineAM:

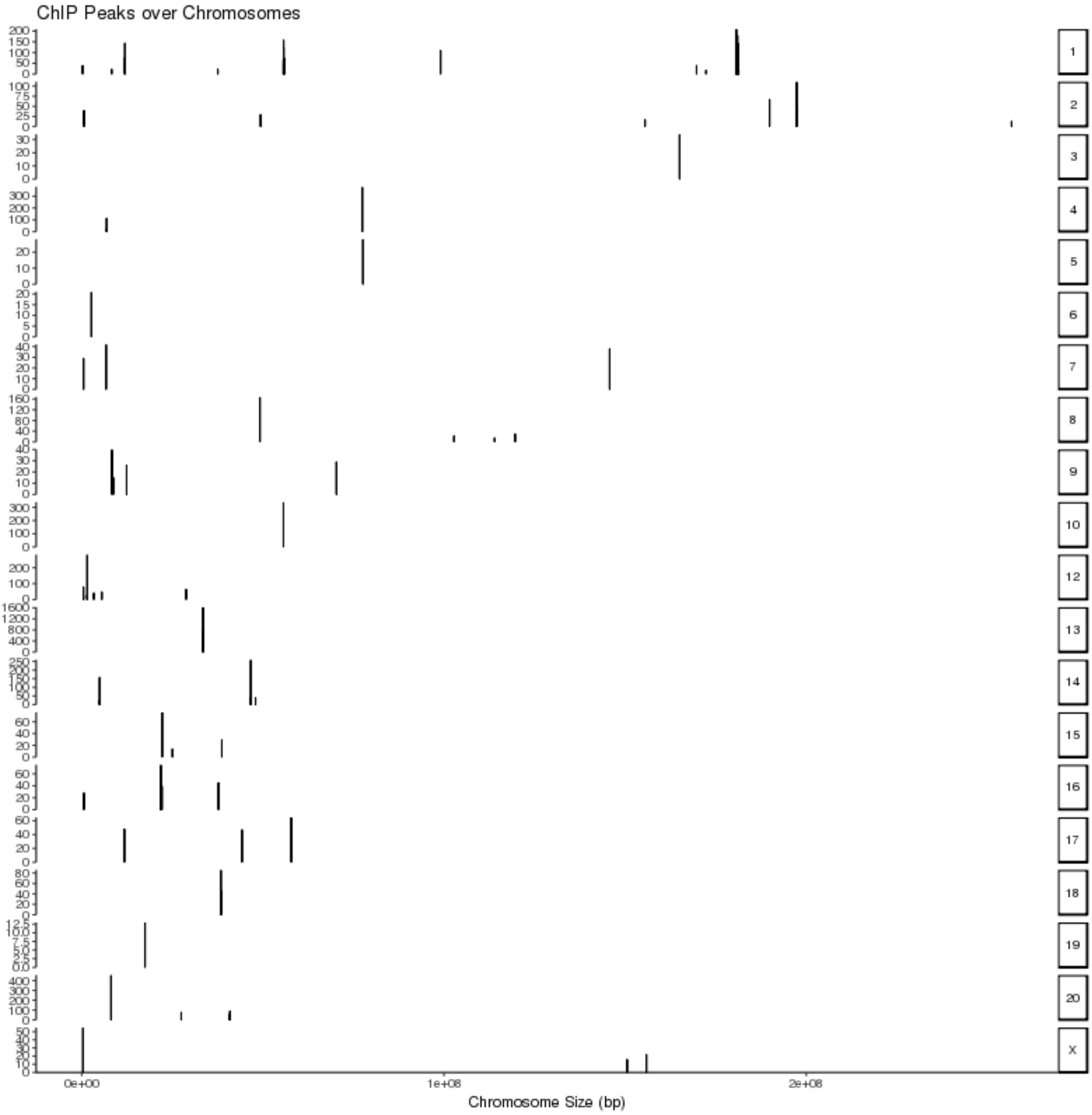

Replicate G-MR-baselineAM:

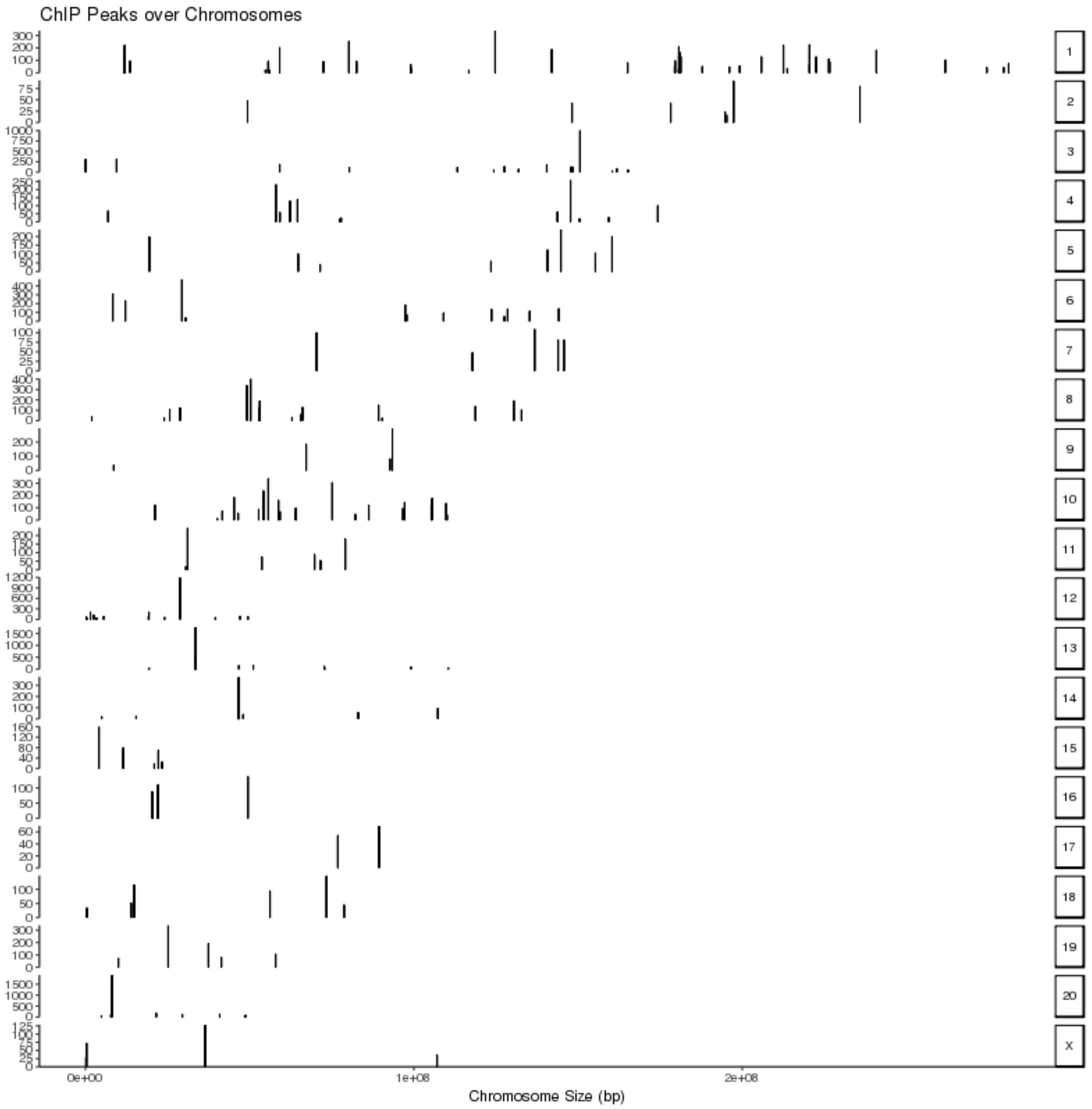

Replicate I-GR-baselinePM:

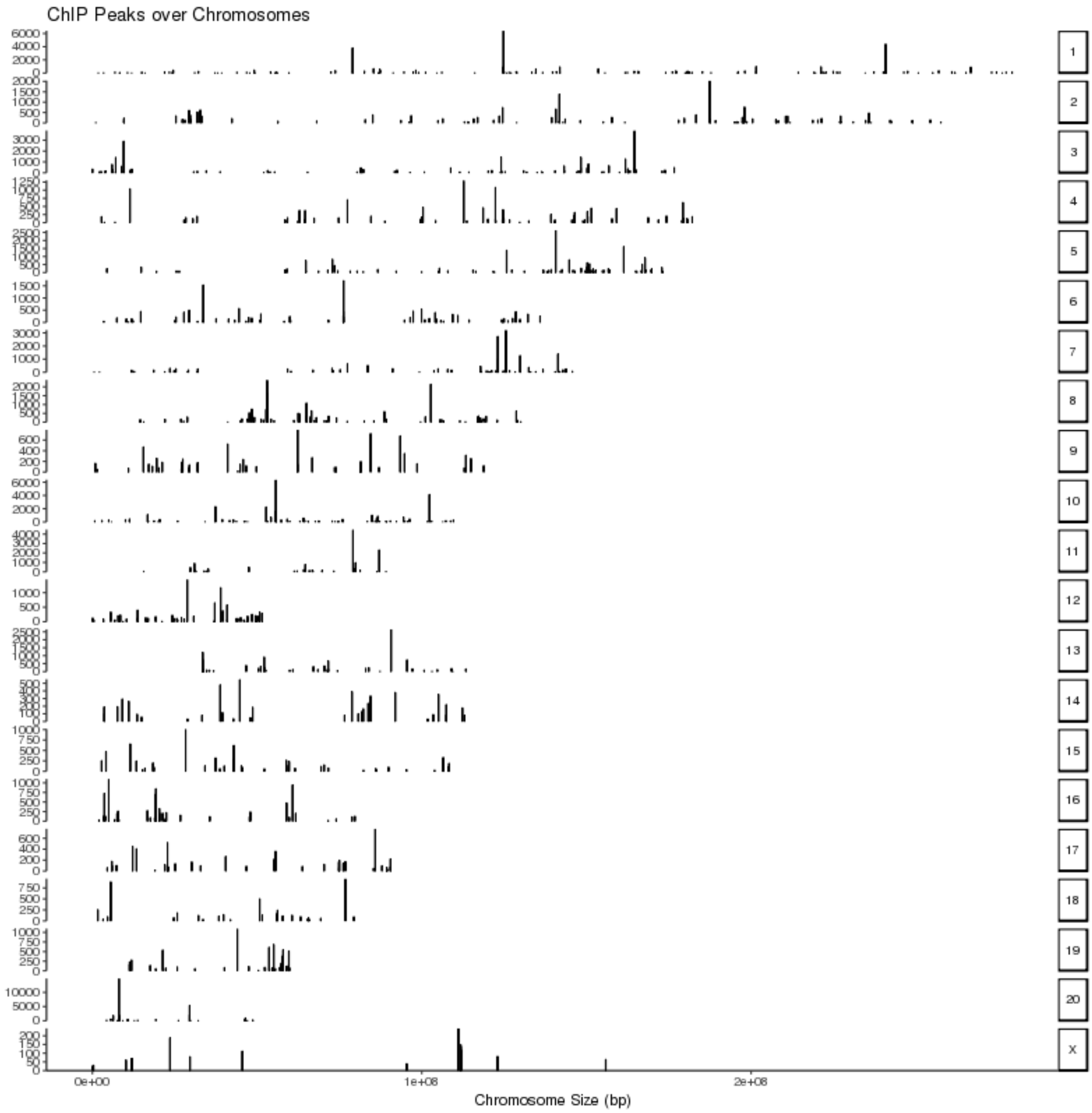

Replicate I-MR-baselinePM:

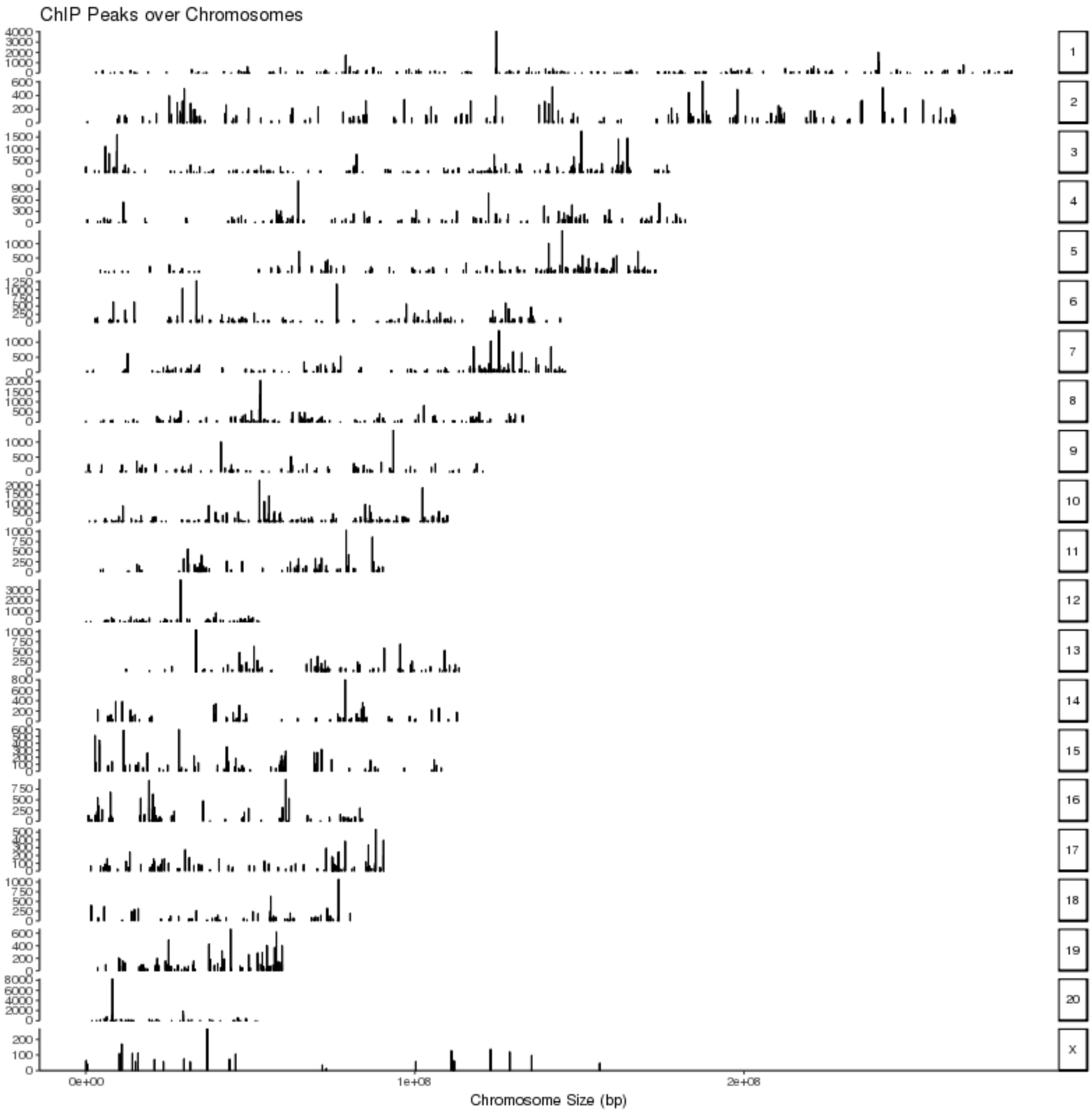

Replicate K-GR-fs30:

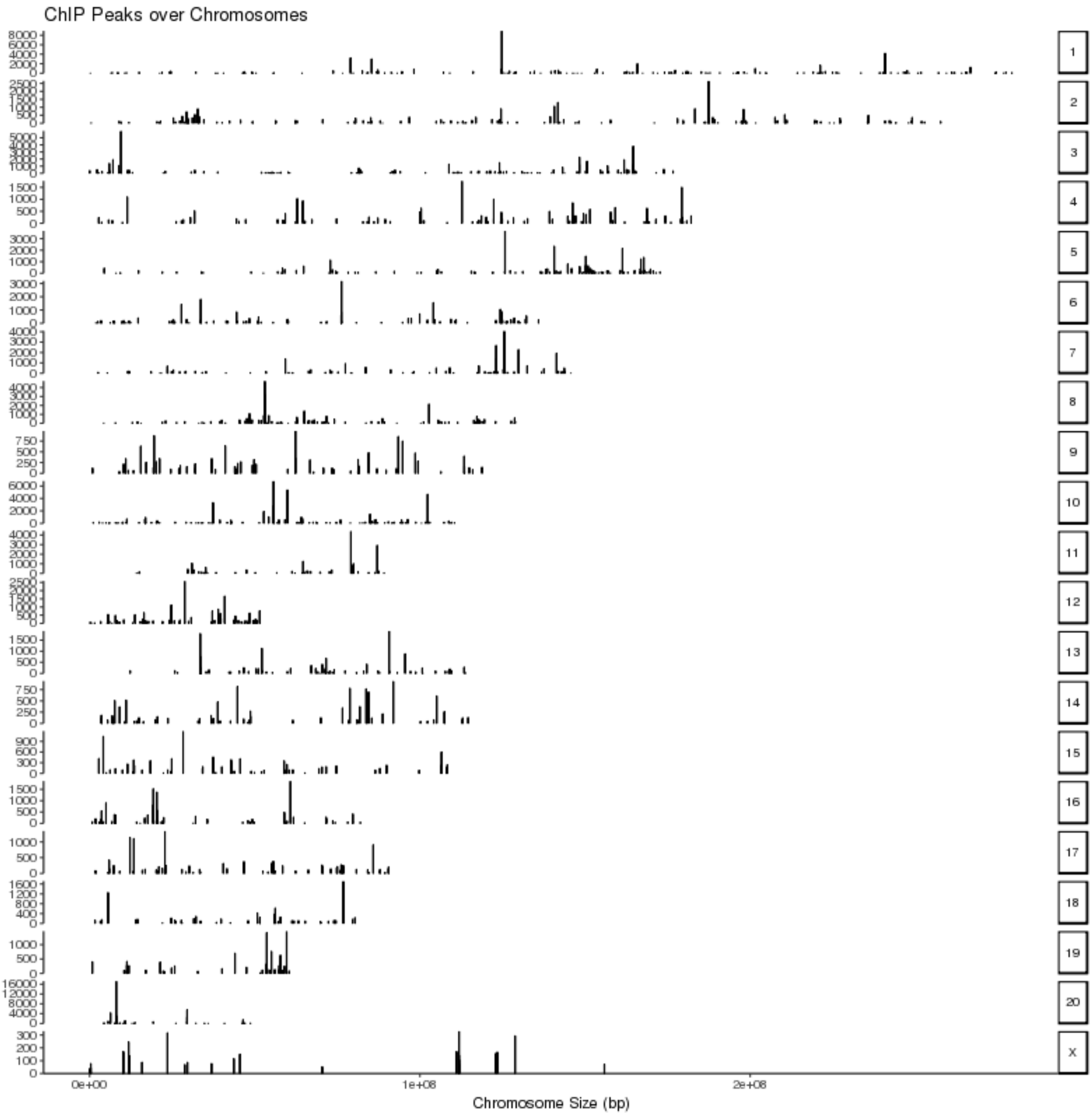

Replicate K-MR-fs30:

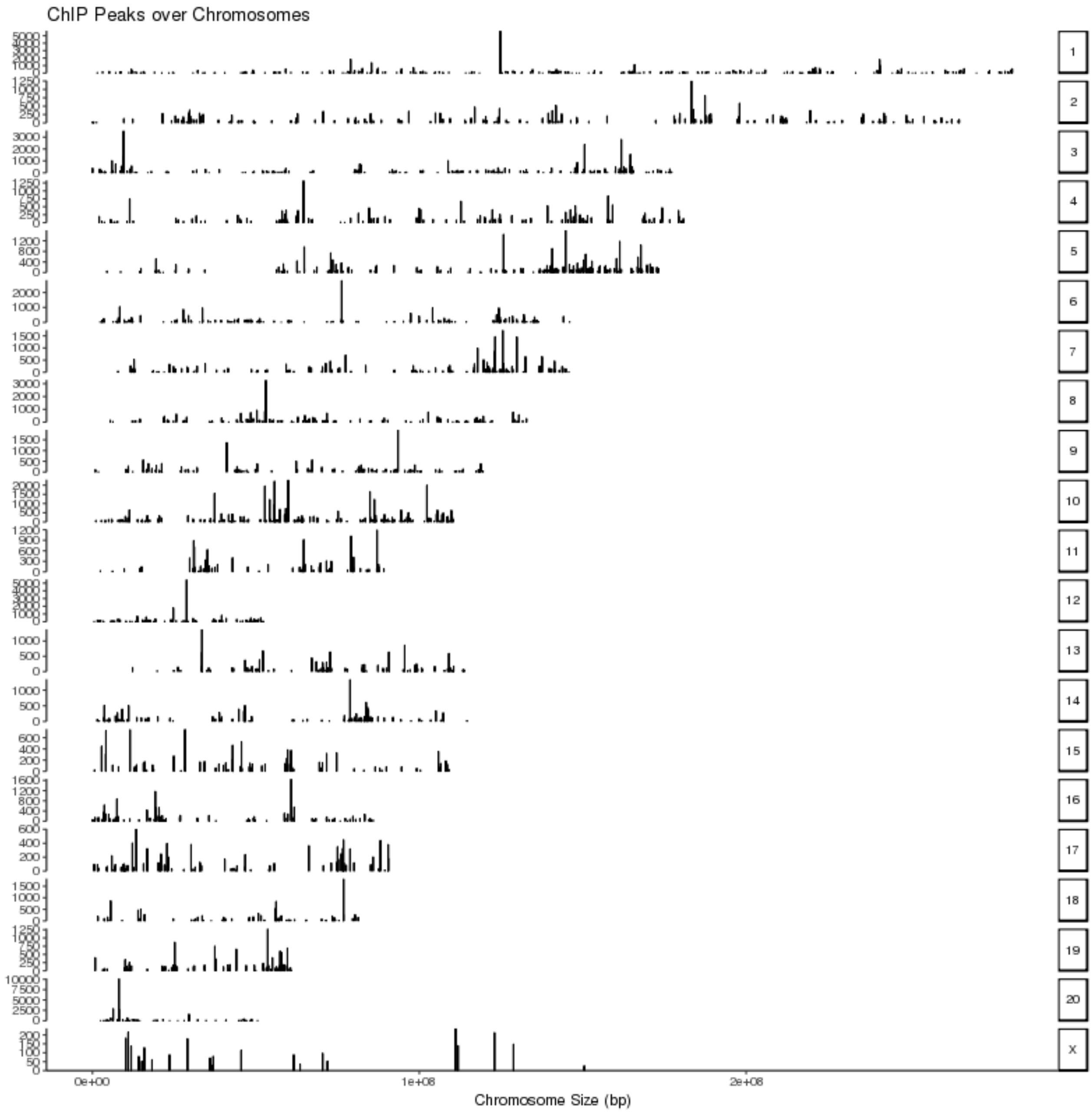

Replicate P-GR-baselineAM:

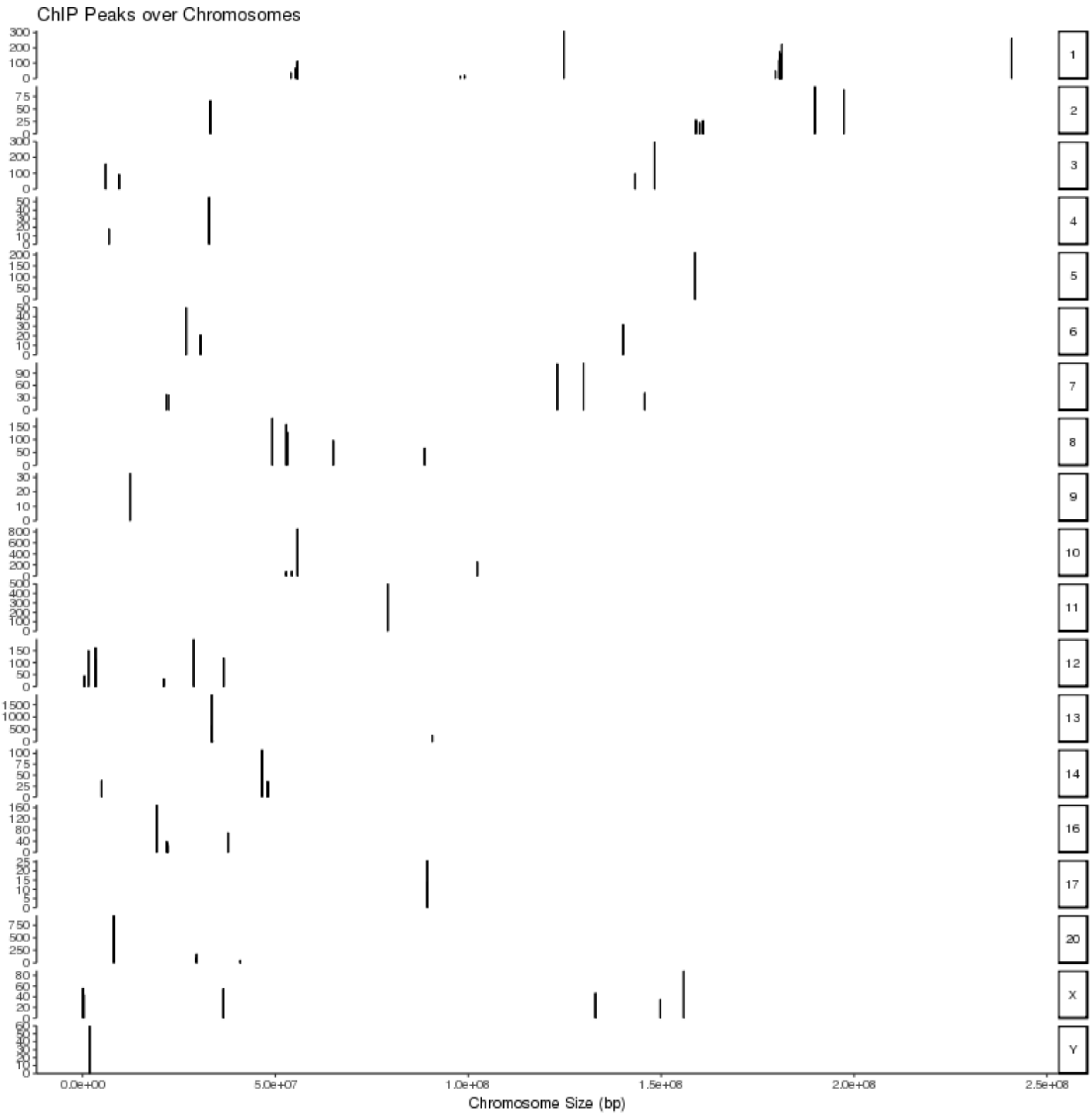

Replicate P-MR-baselineAM:

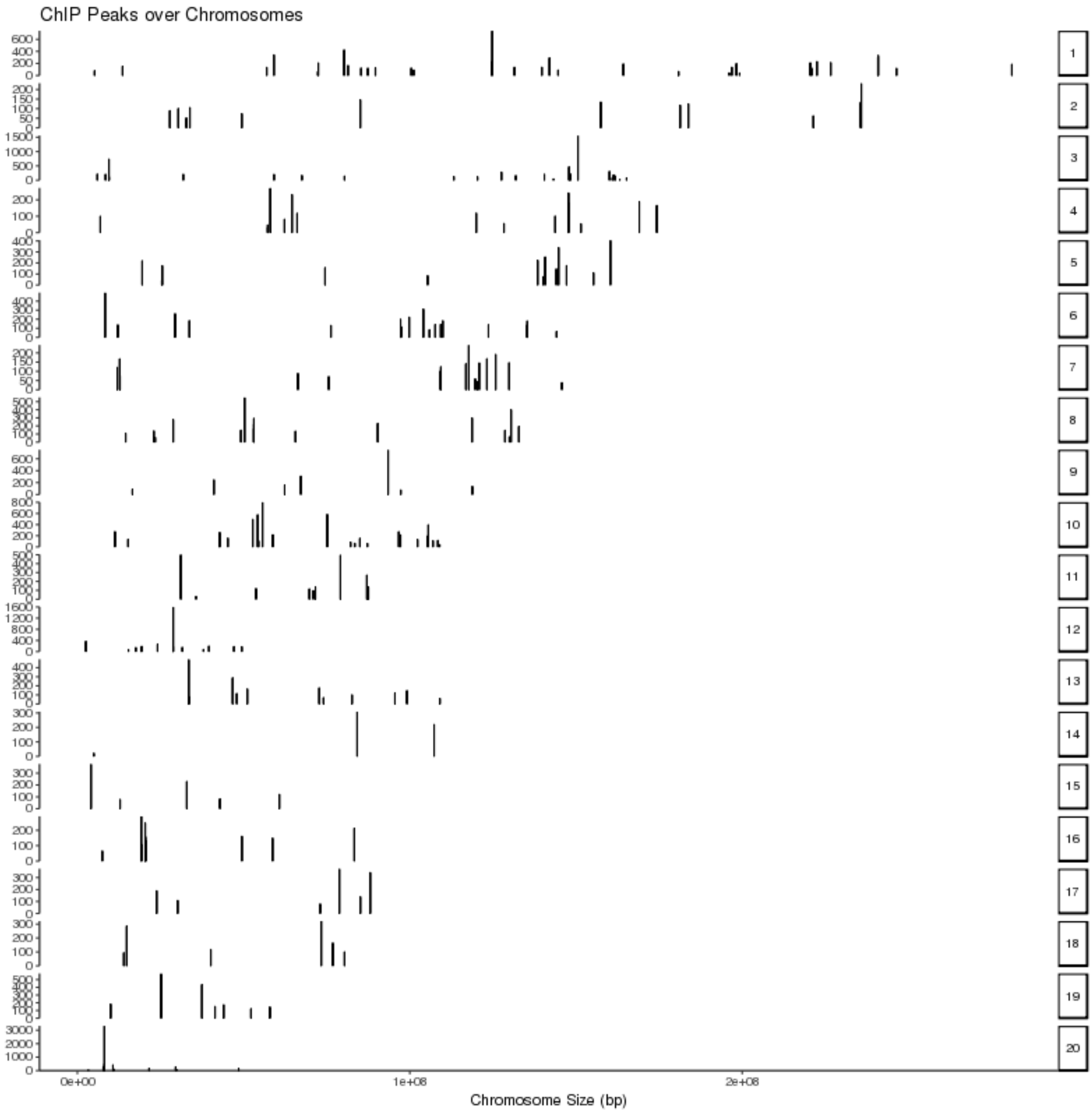

Replicate Q-GR-fs30:

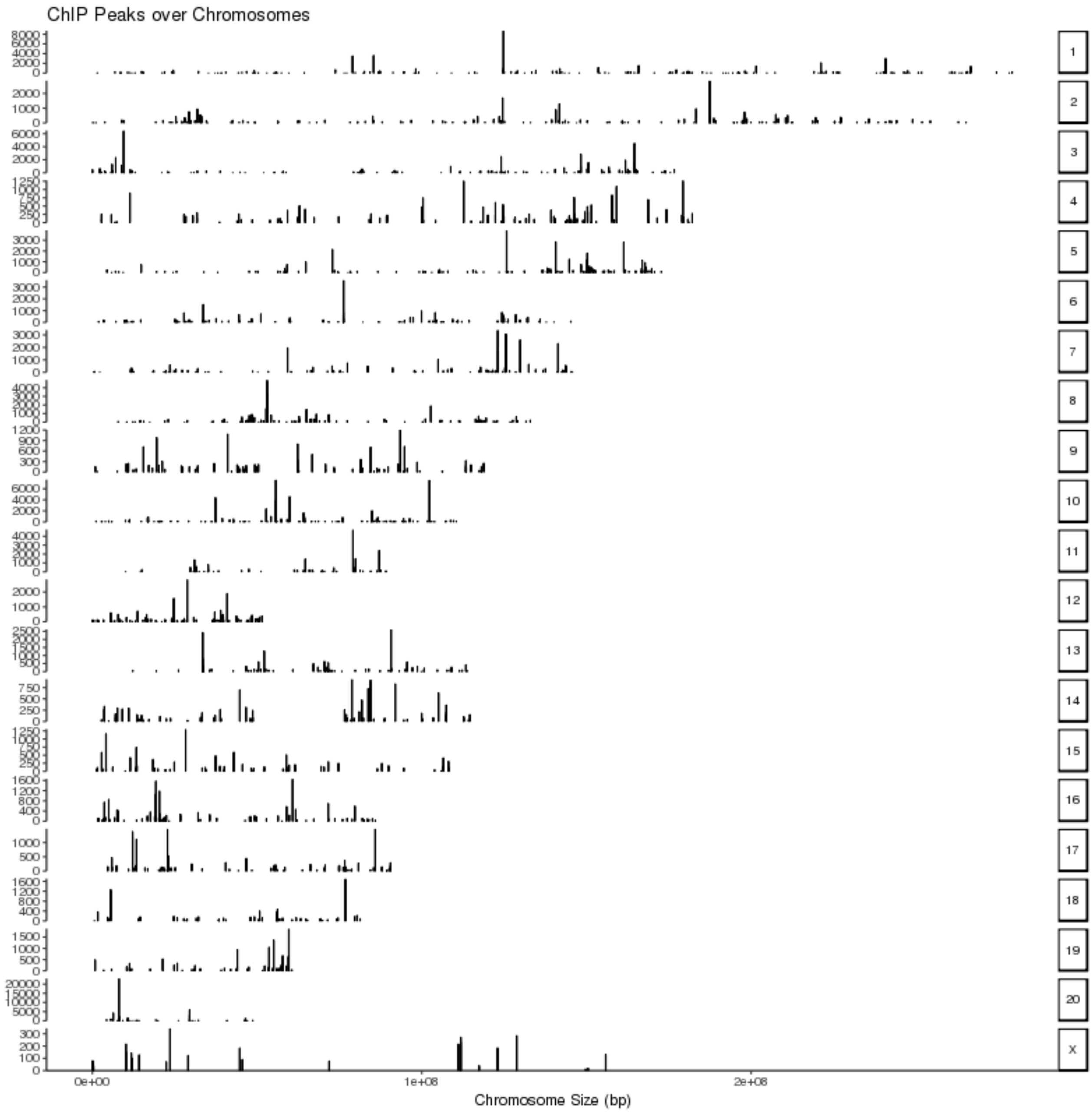

Replicate Q-MR-fs30:

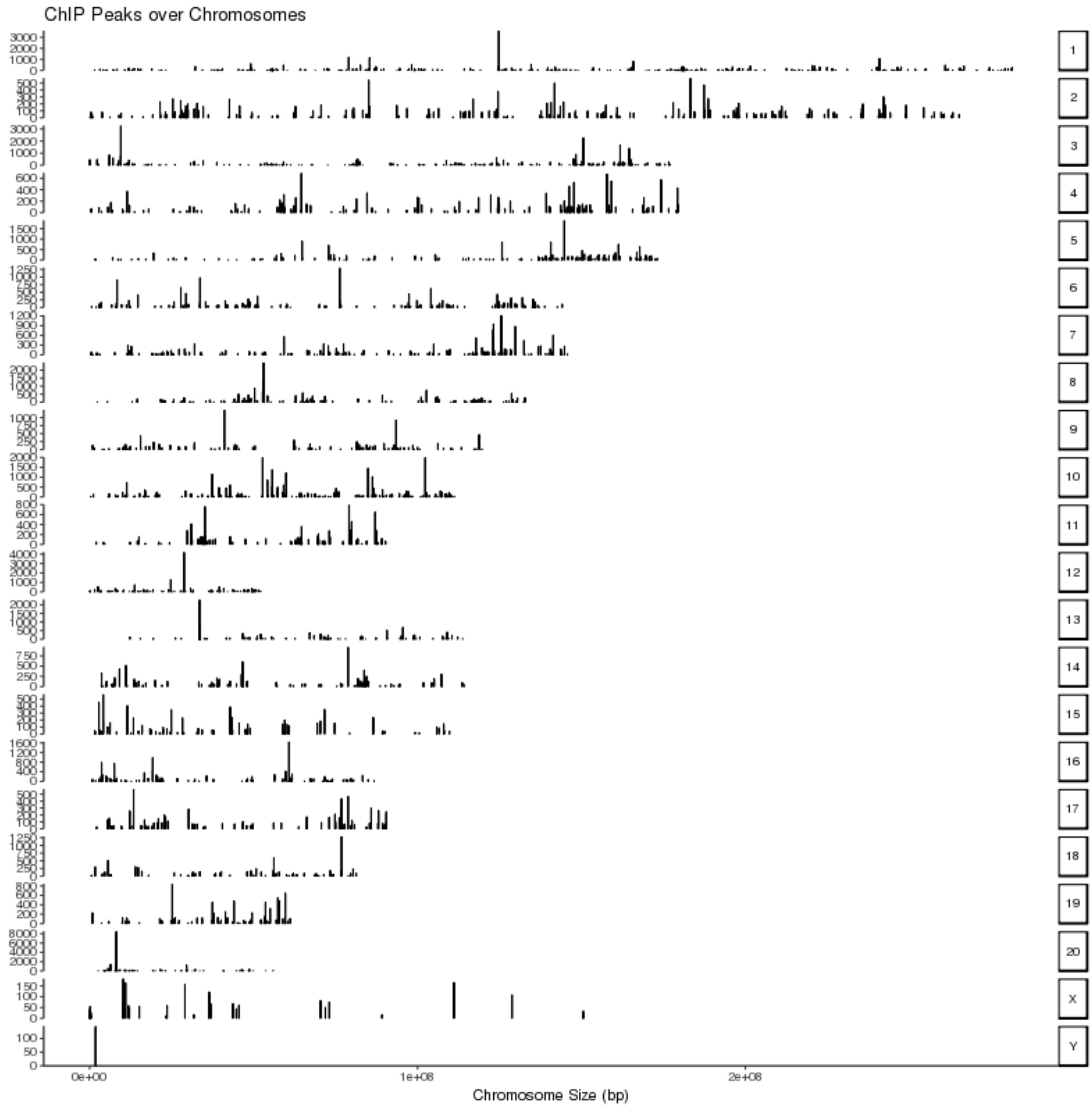

Replicate R-GR-baselinePM:

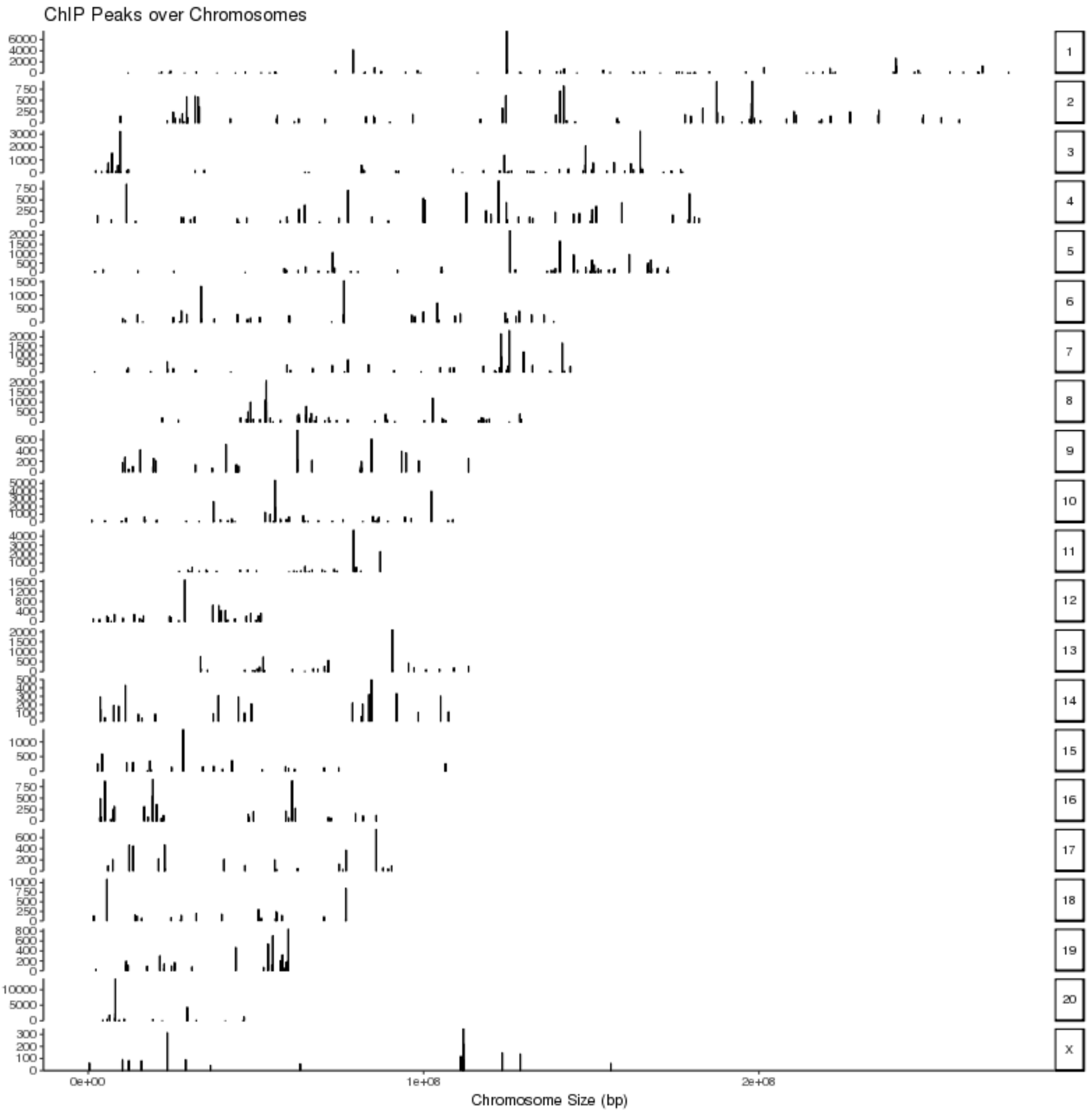

Replicate R-MR-baselinePM:

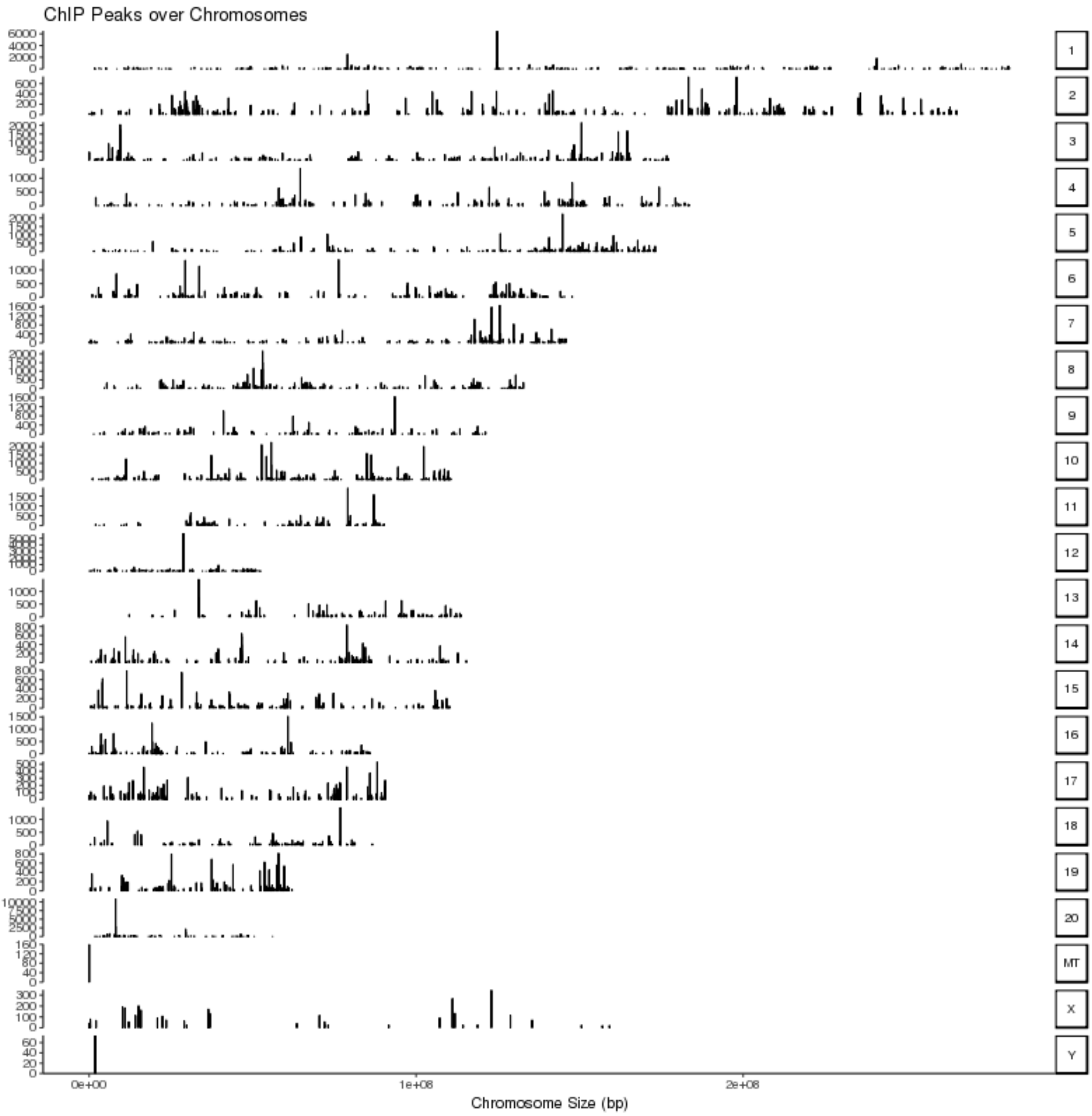

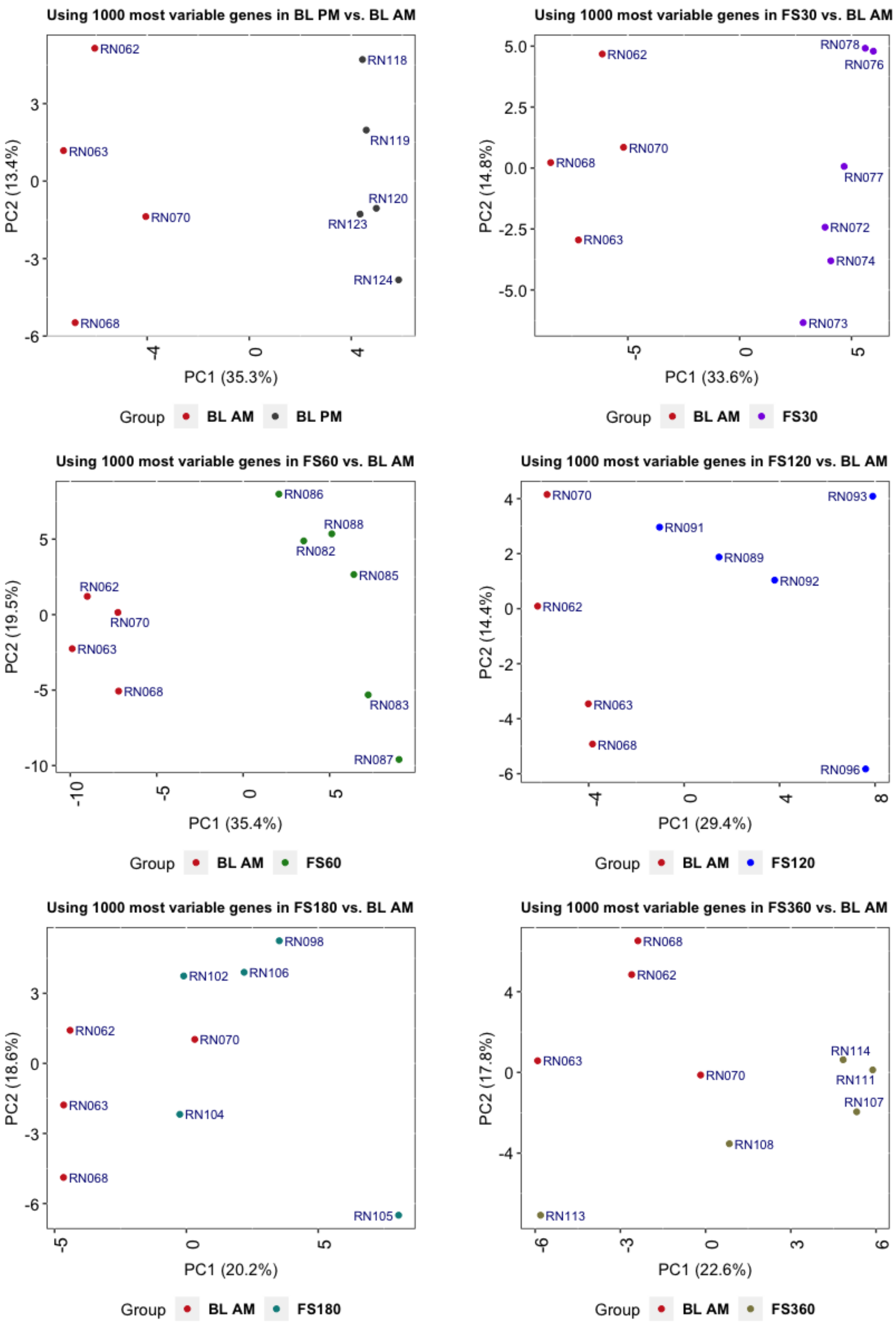

Supplementary Fig. 3: Principal component analysis (PCA) of the top-1000 most variable genes based on intronic read data. This figure shows all PCA plots including the ones presented in Figure 3 to allow comparison of all plots within one figure. Limited clustering is evident in FS120 vs BLAM, FS180 vs BLAM, FS360 vs BLAM supporting the reduction in genes responding significantly to stress at these Time points compared with other comparisons (BLPM vs BLAM, FS30 vs BLAM, FS60 vs BLAM; this figure and Fig. 3 a-c).

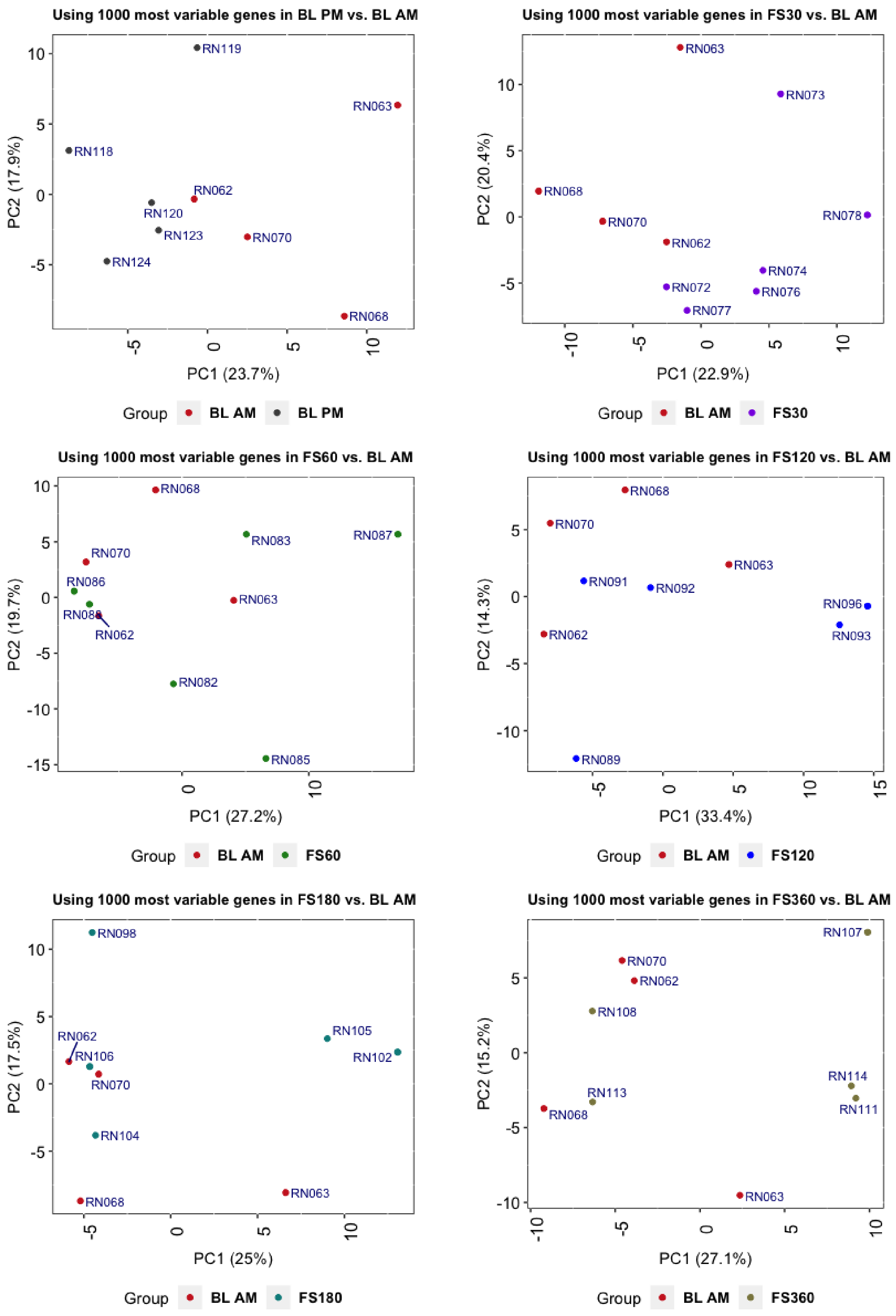

Supplementary Fig. 4: Principal component analysis (PCA) of the top 1000 most variable genes based on exonic read data. Clustering of samples by group is largely absent along either eigenvector (PC1 or PC2) indicating that much of the variance in the data cannot be explained by group differences.

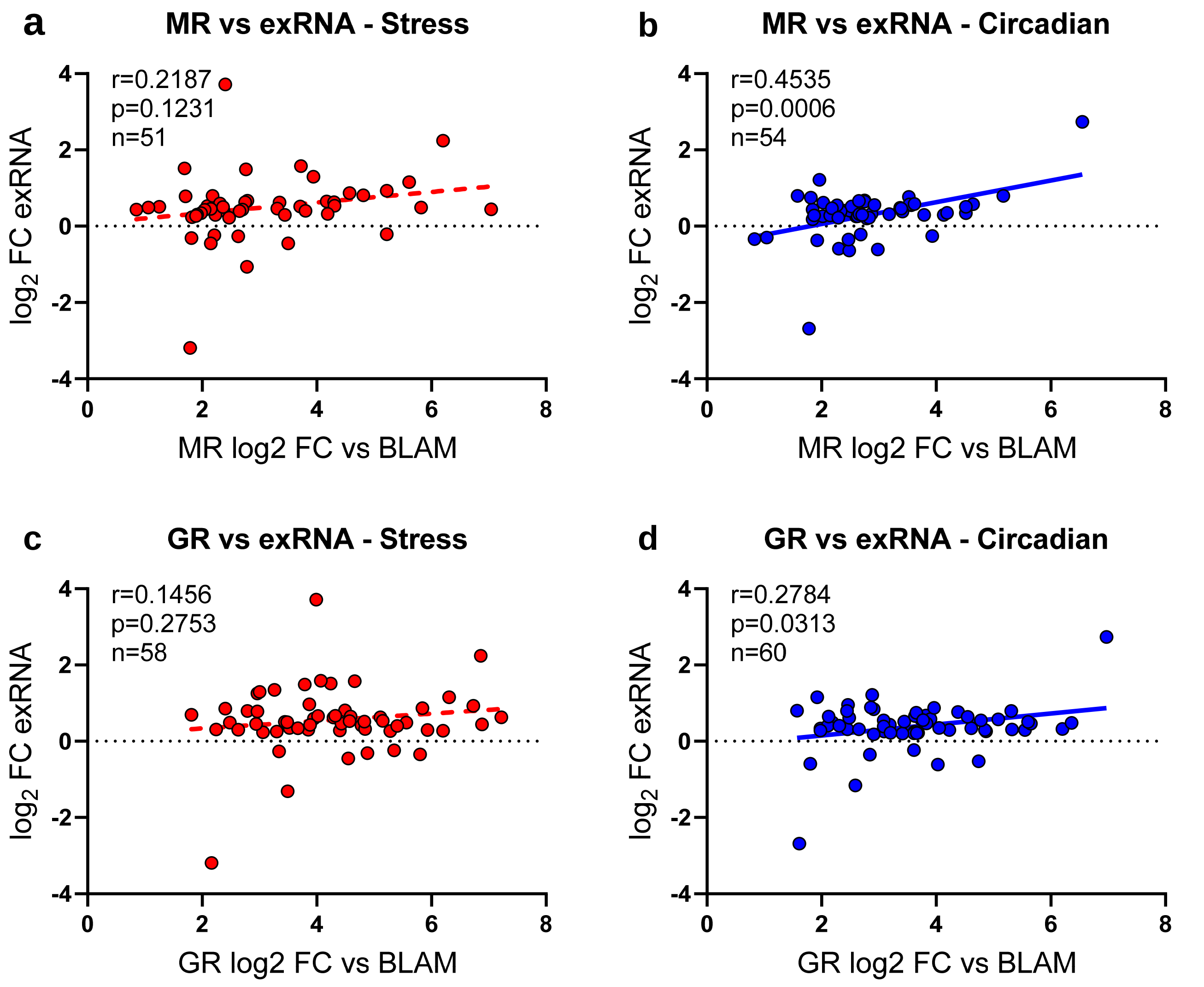

Supplementary Fig. 5: Spearman rank correlation analysis between the fold-change in receptor binding and the associated fold-change in RNA expression under stress and circadian changes. **a**, Correlation between MR binding and exRNA under stress conditions; **b**, Correlation between MR binding and exRNA under circadian conditions; **c**, Correlation between GR binding and exRNA under stress conditions; **d**, Correlation between GR binding and exRNA under circadian conditions. The results of the correlation analysis are depicted in the subfigures. For more information and correlation statistical analyses, see text of the Results section. Source data are provided in the Source Data File.

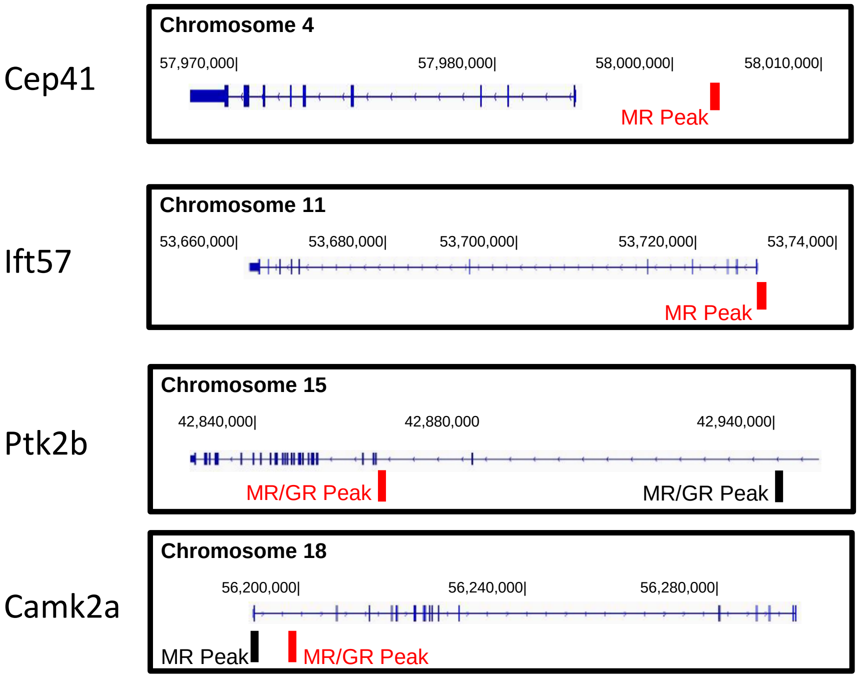

Supplementary Fig. 6: Gene maps showing location of MR and GR binding peaks in the rat genome based on our MR and GR ChIP-Seq data. An MR binding peak is situated within the promoter of the cilia-related genes *Cep41* and *Ift57*. The synaptic plasticity-related genes *Ptk2b* and *Camk2a* contain multiple stress- and circadian-responsive MR and GR peaks. The locations depicted in red are the ones targeted by primers designed against MR and/or GR binding regions within these genes concerning which data are shown in Suppl. Figs 7 & 9. Gene tracks were adapted from the Rnor_6.0 assembly (genome.ucsc.edu/)

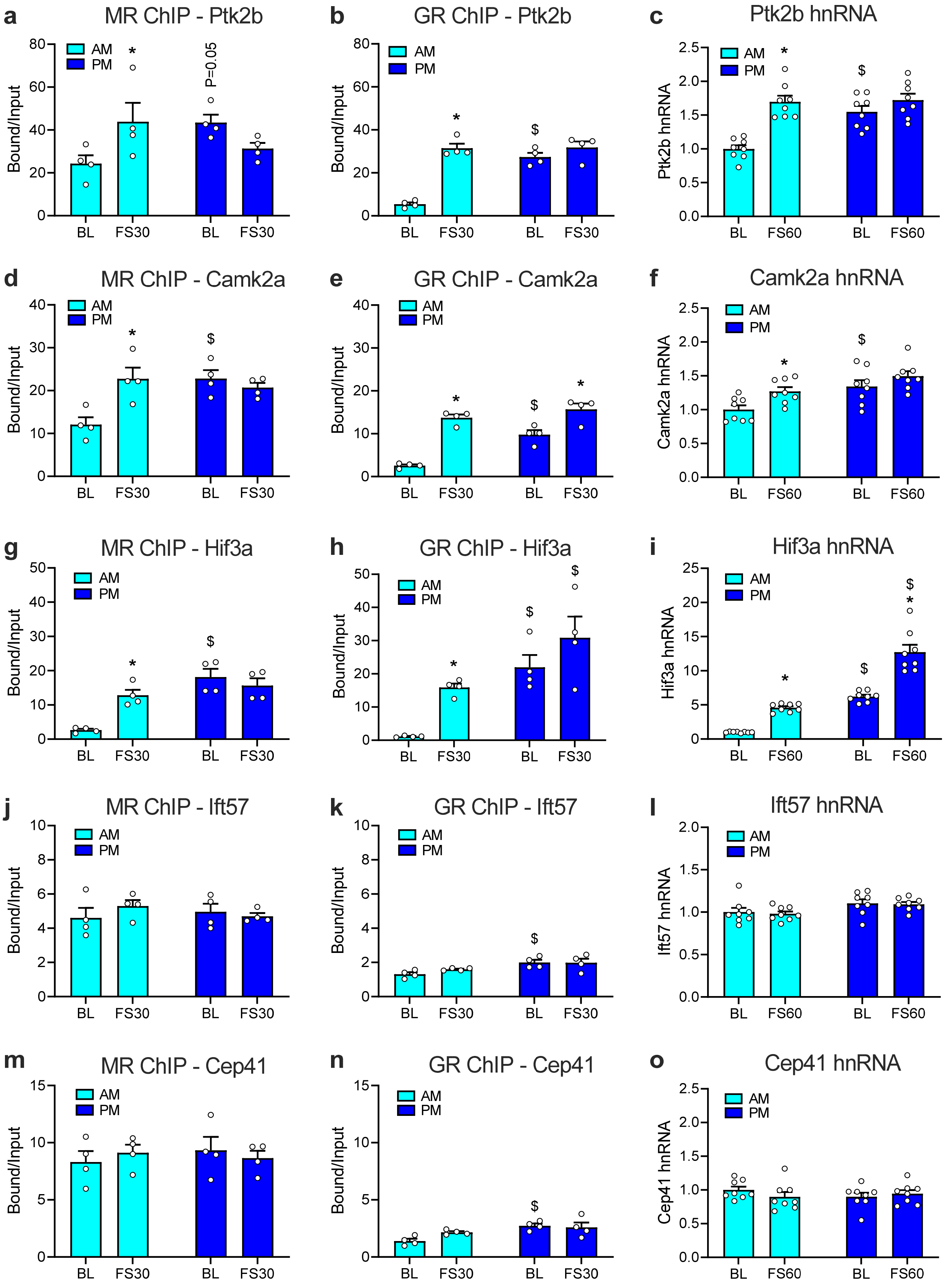

The legend of Supplementary Figure 7 is on the next page.

Supplementary Fig. 7: Validation of ChIP-Seq and RNA-Seq findings in an independent cohort of rats killed under baseline (BL) conditions or 30 min (for ChIP) or 60 min (for hnRNA) after exposure to FS performed during the early morning circadian trough (AM) or late afternoon circadian peak (PM). ChIP-qPCR and RNA analysis were conducted on the genes *Ptk2b* (**a,** MR ChIP; **b,** GR ChIP**; c,** hnRNA), *Camk2a* (**d**, MR ChIP; **e**, GR ChIP; **f**, hnRNA), *Hif3a* (**g**, MR ChIP; **h**, GR ChIP; **i**, hnRNA), *Ift57* (**j**, MR ChIP; **k**, GR ChIP; **l**, hnRNA) and *Cep41* (**m**, MR ChIP; **n**, GR ChIP; **o**, hnRNA). The MR and GR ChIP graphs show mean enrichment ((Bound/Input (B/I); mean ± SEM; n=4). The hnRNA graphs show the results from hnRNA RT-qPCR analyses (mean ± SEM, n=8-9). Statistical analysis: Two-way ANOVA: (**a**) MR ChIP – Ptk2b; effect of AM/PM: $F(1, 12)=0.3773$, $p=0.55$; effect of Group: $F(1, 12)=0.4682$, $p=0.51$; interaction AM/PM x Group: $F(1, 12)=8.728$, $p=0.01$ (**b**) GR ChIP – Ptk2b; effect of AM/PM: $F(1, 12)=29.40$, $p<0.001$; effect of Group: $F(1, 12)=54.81$, $p<0.001$; interaction AM/PM x Group: $F(1, 12)=27.71$, $p<0.001$ (**c**) Ptk2b hnRNA; effect of AM/PM: $F(1, 28)=9.153$, $p=0.0053$; effect of Group: $F(1, 28)=20.76$, $p<0.0001$; interaction AM/PM x Group: $F(1, 28)=6.897$, $p<0.0138$ (**d**) MR ChIP – Camk2a; effect of AM/PM: $F(1, 12)=5.040$, $p=0.0444$; effect of Group: $F(1, 12)=4.834$, $p=0.0483$; interaction AM/PM x Group: $F(1, 12)=10.91$, $p=0.0063$ (**e**) GR ChIP – Camk2a; effect of AM/PM: $F(1, 12)=22.93$, $p=0.0004$; effect of Group: $F(1, 12)=78.97$, $p<0.0001$; interaction AM/PM x Group: $F(1, 12)=7.636$, $p=0.0172$ (**f**) Camk2a hnRNA; effect of AM/PM: $F(1, 28)=14.61$, $p=0.0007$; effect of Group: $F(1, 28)=80.53$, $p=0.0084$; interaction AM/PM x Group: $F(1, 28)=0.6296$, $p<0.4342$ (**g**) MR ChIP – Hif3a; effect of AM/PM: $F(1, 12)=26.45$, $p=0.0002$; effect of Group: $F(1, 12)=4.592$, $p=0.0533$; interaction AM/PM x Group: $F(1, 12)=12.56$, $p=0.004$ (**h**) GR ChIP – Hif3a; effect of AM/PM: $F(1, 12)=23.11$, $p=0.0004$; effect of Group: $F(1, 12)=10.05$, $p=0.0081$; interaction AM/PM x Group: $F(1, 12)=0.6184$, $p=0.4469$ (**i**) Hif3a hnRNA; effect of AM/PM: $F(1, 28)=139.2$, $p<0.0001$; effect of Group: $F(1, 28)=79.43$, $p<0.0001$; interaction AM/PM x Group: $F(1, 28)=6.665$, $p<0.0154$ (**j**) MR ChIP – Ift57; effect of AM/PM: $F(1, 12)=0.2445$, $p=0.6299$; effect of Group: $F(1, 12)=0.07847$, $p=0.7842$; interaction AM/PM x Group: $F(1, 12)=1.254$, $p=0.2847$ (**k**) GR ChIP – Ift57; effect of AM/PM: $F(1, 12)=11.97$, $p=0.0047$; effect of Group: $F(1, 12)=0.7770$, $p=0.3954$; interaction AM/PM x Group: $F(1, 12)=0.9073$, $p=0.3596$ (**l**) Ift57 hnRNA; effect of AM/PM: $F(1, 28)=4.457$, $p=0.0438$; effect of Group: $F(1, 28)=0.2571$, $p=0.6161$; interaction AM/PM x Group: $F(1, 28)=0.2566$, $p<0.06164$ (**m**) MR ChIP – Cep41; effect of AM/PM: $F(1, 12)=0.004168$, $p=0.9496$; effect of Group: $F(1, 12)=0.09523$, $p=0.7629$; interaction AM/PM x Group: $F(1, 12)=0.7050$, $p=0.4175$ (**n**) GR ChIP – Ift57; effect of AM/PM: $F(1, 12)=11.22$, $p=0.0058$; effect of Group: $F(1, 12)=1.354$, $p=0.2672$; interaction AM/PM x Group: $F(1, 12)=2.903$, $p=0.1141$ (**o**) Ift57 hnRNA; effect of AM/PM: $F(1, 28)=0.2079$, $p=0.6519$; effect of Group: $F(1, 28)=0.2442$, $p=0.6250$; interaction AM/PM x Group: $F(1, 28)=2.078$, $p=0.1606$. *Post-hoc* Bonferroni multiple comparisons test: *, $p<0.05$, compared with respective baseline group; $^{\$}$, $p<0.05$, compared with respective AM group. Source data are provided in the Source Data File.

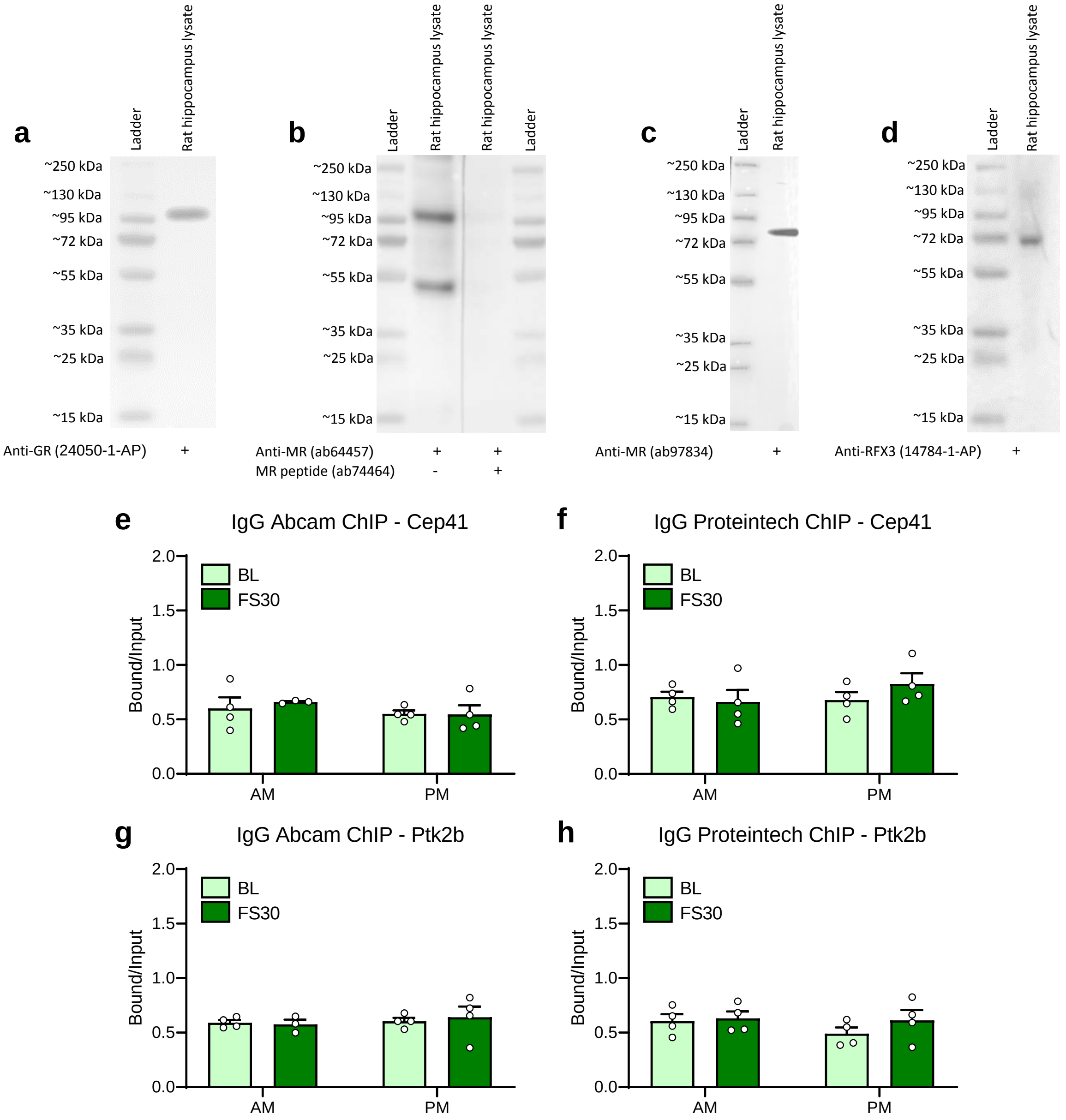

Supplementary Fig. 8. Demonstration of specific immunoreactivity of antibodies used in the validation experiments by Western blotting and non-specific IgG ChIP. Hippocampal whole-cell extracts from male Wistar rats were subjected to SDS-PAGE, electrophoretically transferred to PVDF membrane and incubated with antibody to confirm antibody specificity (n=1, as confirmation only). According to UniProt, GR has two protein isoforms (A, ~87 kDa, P06536-1; B ~84 kDa, P06536-2), MR has three possible isoforms (1, ~106 kDa, P22199-1; 2, ~107 kDa, P22199-2; 3, ~86 kDa P22199-3) and RFX3 sequence remains unreviewed. **a**, anti-GR antibody (rabbit polyclonal, Proteintech, 24050-1-AP), one band >95 kDa marker consistent with the reported GR band (~94-97 kDa, Proteintech); **b**, anti-MR antibody (rabbit polyclonal, Abcam, ab64457), predominant bands at >95 kDa consistent with the reported MR band (~100 kDa, Abcam) and 45 kDa. All bands blocked in the presence of blocking peptide ab74464 confirming MR specificity; **c**, anti-MR antibody (rabbit polyclonal, Abcam, ab97834), one band present ~85 kDa consistent with MR isoform 3 (predicted ~86 kDa, P22199-3 UniProt); **d**, anti-RFX3 antibody (rabbit polyclonal, Proteintech, 14784-1-AP), one band present ~70 kDa consistence with the reported RFX3 band (70-79 kDa, Proteintech). Assessment of non-specific IgG binding in hippocampal chromatin of BL and FS30 male Wistar rats, as in Suppl. Fig. 7) using Abcam IgG (ab171870) and Proteintech IgG (30000-0-AP) at Cep41 (**e**, **f**) and Ptk2b (**g**, **h**). Enrichment (Mean ± SEM, n=3-4 biologically independent repeats; see dots for exact number) was <1 in all cases with no significant differences between groups. Statistical analysis: Two-way ANOVA: (**e**) Abcam IgG ChIP – Cep41; effect of Group: $F(1, 11)=1.273$, $p=0.2832$; effect of AM/PM: $F(1, 11)=0.1372$, $p=0.7181$; interaction Group x AM/PM: $F(1, 11)=0.1823$, $p=0.6776$. (**f**) Proteintech IgG ChIP – Cep41; effect of Group: $F(1, 12)=0.6452$, $p=0.4374$; effect of AM/PM: $F(1, 12)=0.3582$, $p=0.5606$; interaction Group x AM/PM: $F(1, 12)=1.250$, $p=0.2854$. (**g**) Abcam IgG ChIP – Ptk2b; effect of Group: $F(1, 11)=0.4162$, $p=0.5321$; effect of AM/PM: $F(1, 11)=0.02765$, $p=0.8710$; interaction Group x AM/PM: $F(1, 11)=0.1810$, $p=0.6787$. (**h**) Proteintech IgG ChIP – Ptk2b; effect of Group: $F(1, 12)=0.8787$, $p=0.3670$; effect of AM/PM: $F(1, 12)=1.048$, $p=0.3261$; interaction Group x AM/PM: $F(1, 12)=0.4542$, $p=0.5131$. Source data are provided as a Source Data file.

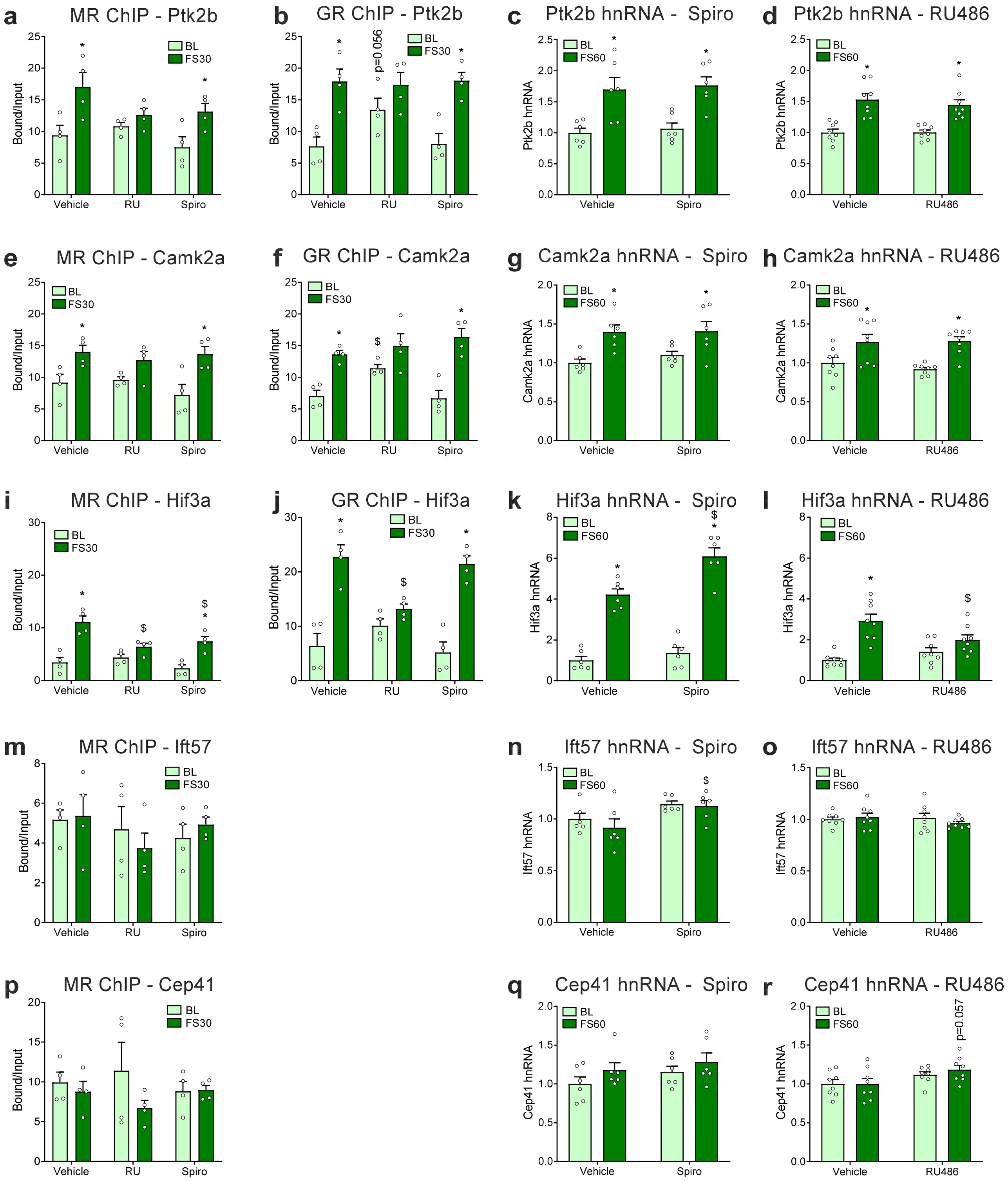

The legend of Supplementary Figure 9 is on the next page.

Supplementary Fig. 9: Effect of MR and GR antagonists on hippocampal MR and GR binding and RNA transcription in response to stress or circadian changes. The MR antagonist spironolactone (Spiro, 50 mg/kg), GR antagonist RU486 (RU, 100 mg/kg) or vehicle (50% condensed milk) was administered to rats via voluntary dosing (1 ml/rat, see Methods for details). Rats were subsequently killed under baseline (BL) conditions or 30 min (for ChIP; FS30) or 60 min (for hnRNA; FS60) after exposure to FS. ChIP-qPCR and RNA analysis were conducted on the genes *Ptk2b* (**a,** MR ChIP; **b,** GR ChIP; **c** & **d,** hnRNA), *Camk2a* (**e**, MR ChIP; **f**, GR ChIP; **g** & **h**, hnRNA), *Hif3a* (**i**, MR ChIP; **j**, GR ChIP; **k** & **l** hnRNA), *Ift57* (**m**, MR ChIP; **n & o**, hnRNA) and *Cep41* (**p**, MR ChIP; **q** & **r**, hnRNA). GR ChIP samples were not analysed for Ift57 and Cep41 due to poor GR enrichment levels within these genes shown previously (Suppl. Fig. 7k & 7n). The MR and GR ChIP graphs show mean enrichment ((Bound/Input (B/I); mean ± SEM; n=4 biologically independent replicates). The hnRNA graphs show the results from hnRNA RT-qPCR analyses (mean ± SEM, n=6-8 biologically independent replicates; see dots in graphs for exact n of each experimental group). Statistical analysis: Two-way ANOVA: (**a**) MR ChIP – Ptk2b; effect of Antagonist: F(2,18)=1.823, p=0.1898; effect of Group: F(1,18)=16.65, p=0.0007; interaction Antagonist x Group: F(2,18)=1.926, p=0.1746 (**b**) GR ChIP – Ptk2b; effect of Antagonist: F(2,18)=1.398, p=0.1415; effect of Group: F(1,18)=33.24, p<0.0001; interaction Antagonist x Group: F(2,18)=2.184, p=0.1415 (**c**) Ptk2b hnRNA – Spiro; effect of Antagonist: F(1,20)=0.2319, p=0.6353; effect of Group: F(1,20)=26.68, p<0.0001; interaction Antagonist x Group: F(1,20)=1.757e^{-5}, p<0.9967 (**d**) Ptk2b hnRNA – RU; effect of Antagonist: F(1,28)=0.3550, p=0.5561; effect of Group: F(1,28)=46.07, p<0.0001; interaction Antagonist x Group: F(1,28)=0.3800, p=0.5426 (**e**) MR ChIP – Camk2a; effect of Antagonist: F(2,18)=0.4317, p=0.6560; effect of Group: F(1,18)=21.95, p=0.0002; interaction Antagonist x Group: F(2,18)=0.8952, p=0.4260 (**f**) GR ChIP – Camk2a; effect of Antagonist: F(2,18)=2.927, p=0.0793; effect of Group: F(1,18)=45.95, p<0.0001; interaction Antagonist x Group: F(2,18)=3.297, p=0.0603 (**g**) Camk2a hnRNA – Spiro; effect of Antagonist: F(1,20)=0.4206, p=0.5240; effect of Group: F(1,20)=17.29, p=0.0005; interaction Antagonist x Group: F(1,20)=0.2894, p=0.5965 (**h**) Camk2a hnRNA – RU; effect of Antagonist: F(1,28)=0.3139, p=0.5798; effect of Group: F(1,28)=22.75, p<0.0001; interaction Antagonist x Group: F(1,28)=0.4770, p=0.4955 (**i**) MR ChIP – Hif3a; effect of Antagonist: F(2,18)=4.401, p=0.0278; effect of Group: F(1,18)=50.49, p<0.0001; interaction Antagonist x Group: F(2,18)=5.442, p=0.0142 (**j**) GR ChIP – Hif3a; effect of Antagonist: F(2,18)=1.368, p=0.2799; effect of Group: F(1,18)=68.54, p<0.0001; interaction Antagonist x Group: F(2,18)=9.440, p=0.0016 (**k**) Hif3a hnRNA – Spiro; effect of Antagonist: F(1,20)=13.15, p=0.0017; effect of Group: F(1,20)=168.0, p<0.0001; interaction Antagonist x Group: F(1,20)=6.067, p=0.0230 (**l**) Hif3a hnRNA – RU; effect of Antagonist: F(1,28)=1.208, p=0.2810; effect of Group: F(1,28)=28.03, p<0.0001; interaction Antagonist x Group: F(1,28)=7.839, p=0.0092 (**m**) MR ChIP – Ift57; effect of Antagonist: F(2,18)=0.8694, p=0.4361; effect of Group: F(1,18)=0.0001365, p=0.9709; interaction Antagonist x Group: F(2,18)=0.5416, p=0.5910 (**n**) Ift57 hnRNA – Spiro; effect of Antagonist: F(1,20)=8.842, p=0.0075; effect of Group: F(1,20)=0.7432, p=0.3988; interaction Antagonist x Group: F(1,20)=0.3114, p=0.5830 (**o**) Ift57 hnRNA – RU; effect of Antagonist: F(1,28)=0.4470, p=0.5092; effect of Group: F(1,28)=0.2142, p=0.6471; interaction Antagonist x Group: F(1,28)=1.120, p=0.2990 (**p**) MR ChIP – Cep41; effect of Antagonist: F(2,18)=0.03626, p=0.9645; effect of Group: F(1,18)=1.701, p=0.2086; interaction Antagonist x Group: F(2,18)=0.9900, p=0.3909 (**q**) Cep41 hnRNA – Spiro; effect of Antagonist: F(1,20)=1.797, p=0.1951; effect of Group: F(1,20)=2.570, p=0.1246; interaction Antagonist x Group: F(1,20)=0.05927, p=0.8101 (**r**) Cep41 hnRNA – RU; effect of Antagonist: F(1,28)=7.079, p=0.0128; effect of Group: F(1,28)=0.3142, p=0.5796; interaction Antagonist x Group: F(1,28)=0.3676, p=0.2990. Post-hoc Bonferroni multiple comparisons test: *, p<0.05 compared with respective BL group; $^{\$}$, P<0.05 compared with respective vehicle group. Source data are provided in the Source Data File.

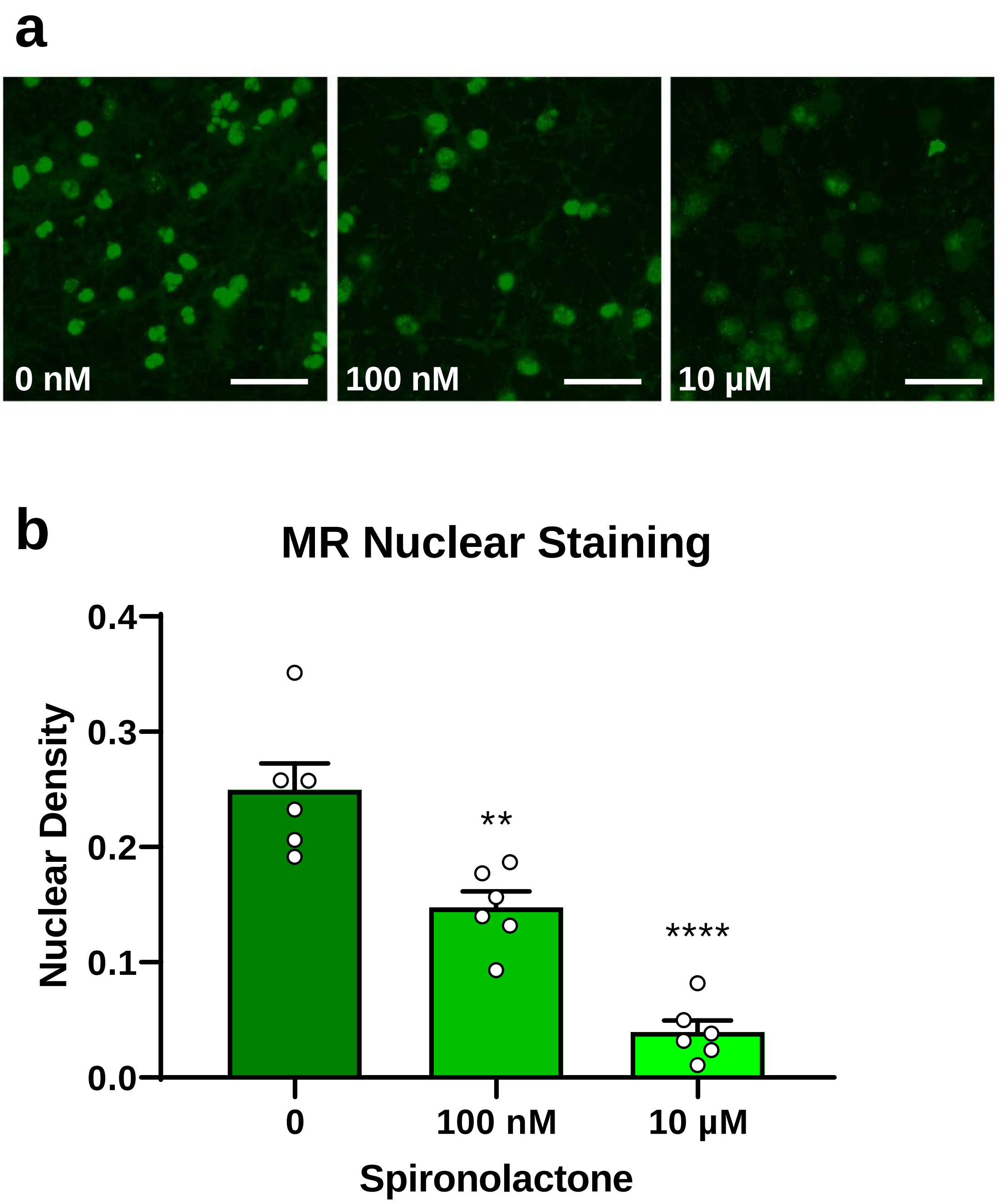

Supplementary Fig. 10. Spironolactone (Spiro) treatment results in decreased nuclear localization of MR in hfNPCs. From the start of neuronal differentiation, hfNPCs were treated for 1 week with 0, 100 nM or 10 μM Spiro after which cells were processed for MR immunocytochemistry (**a**; scale bar = 50μm; see Methods for details). **b**. The integrated nuclear staining density of MR immunoreactivity was assessed using ImageJ with the plugin 'Analyze particles'. Data are expressed as Integrated Nuclear Density (Mean ± SEM, n=6 biologically independent replicates per condition). Oneway ANOVA: $F(2,15)=40.02$, $p<0.0001$; post-hoc Dunnett's test: **, p=0.0011; ****, p<0.0001. Source data are provided in the Source Data File.

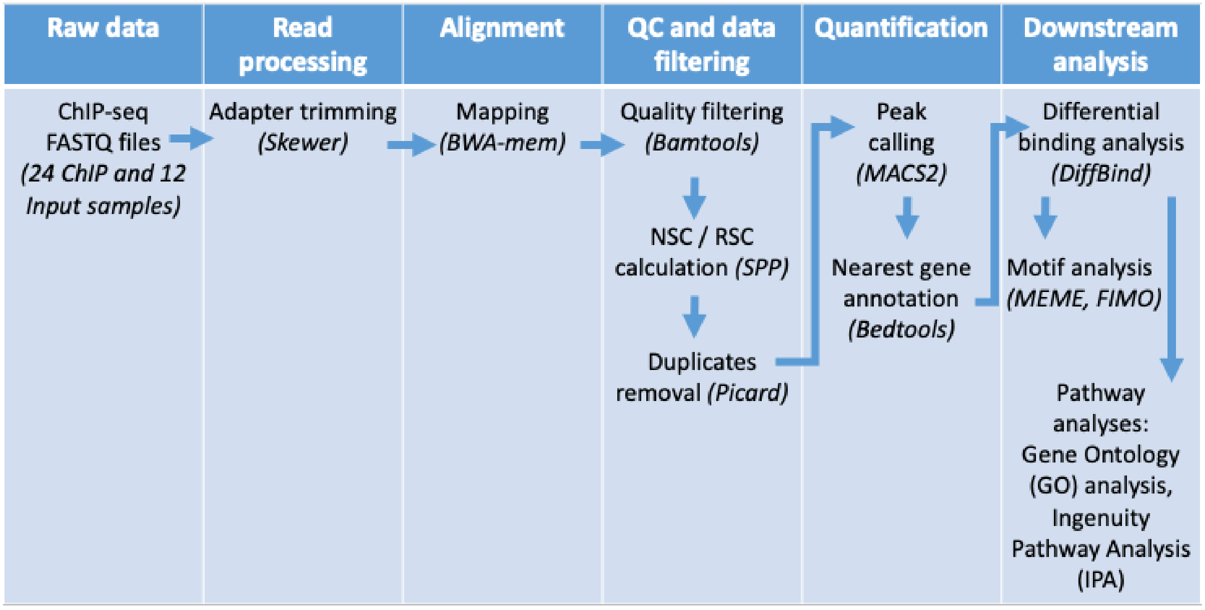

Supplementary Fig. 11. Workflow of the bioinformatic analysis pipeline for our ChIP-Seq data.

Raw data	Alignment	QC metrics	Quantification	Differential Expression (DE)	Pathway analysis
RNAseq FASTQ files *(37 RiboZero RNAseq samples)*	Splice-aware aligner *(Hisat2)*	Picard tools *(CollectRnaSeq Metrics and MarkDuplicates on alignment BAM)*	Quantification to Exon feature GTF or Intron feature GTF and summarized to ENSEMBL gene IDS *(FeatureCounts with parameters* -M -p -O --fraction)<br><br>Normalized read counts for PCA and QC (base R)	Count tables subset for each comparison (*edgeR*)<br>*Step1. Count table normalization*<br>*Step2. Estimate Dispersion and fit data to generalized linear models*<br>*Step 3. Differential expression tested with likelihood ratio tests*	Gene ontology enrichment analysis using DE genes (FDR <0.05) and with length bias correction (*GOseq*)

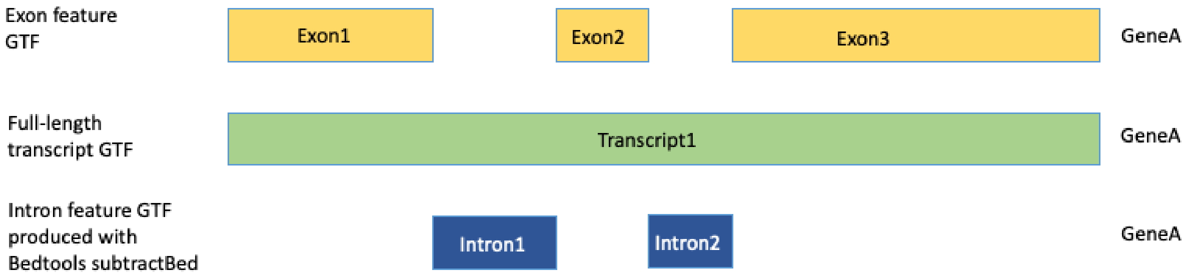

Supplementary Fig. 12. Workflow of the bioinformatic analysis pipeline for our RNA-Seq data.

	MR peaks		GR peaks	
	**Motif**	**% (n=1753)**	**Motif**	**% (n=1066)**
All peaks	GRE	77	GRE	93
	ZNF	76	ZNF	74
	SP	74	SP	70
	KLF	70	KLF	68
	EGR	64	EWSR1-FLI1	44
	**Motif**	**% (n=401)**	**Motif**	**% (n=74)**
Non-GRE containing peaks	SP	80	NR	65
	ZNF	76	STAT	62
	KLF	76	ZNF	51
	EGR	74	PAX	49
	RFX	73	EWSR1-FLI1	48

Supplementary Table 1. FIMO analysis of transcription factor binding motifs within MR and GR binding peaks. The top 5 transcription factor binding motifs found in all MR and GR binding peaks are listed and confirm GRE as the predominant motif. In MR and GR binding peaks without a concensus GRE some variation in top 5 motifs present is observed (i.e. the RFX motif in non-GRE containing MR peaks and the NR, STAT and PAX motifs in non-GRE containing GR peaks).

Target	Gene ID	Accession number	Forward primer sequence (5' - 3')	Reverse primer sequence (5' - 3')	Probe sequence (FAM_5' - 3'_TAMRA)
gDNA	Camk2a	NM_012920	ATTCAGTGTTCCCTTCAACAAACA	CGCCAACCCAGCAGAAGT	TCCGGTTCTGAGGCCTTCTGTGTCCT
	Cep41	NM_001025770	GCCCTTGGCAACCATTGA	TCCCTGTAACCTCTCTTCCGATT	CGGCTTCTCGCTCTCCCTCGCT
	Hif3a	NM_001388512	CTCTCACCGCCTTGCACTAAT	GGGATGGGAGGGCTACAAA	CCACCCCCGCACCCAGACTAAAA
	Ift57	NM_001107093	CTTTGGTTGGCGAGAATTTAGAG	CCCTGTCGCCAACGTTACC	CCATGGTGACCGGCAGCCCT
	Ptk2b	NM_017318	CTTGTCCCCACTTCTGCTACCT	ACAGTGTGCTCTGCCTGATGA	CTCCCACTTCCTCTGTCTCATCTTGCCAA
hnRNA	B-actin	NM_031144	TGTATTCCTTTCTCTACAGATCATGTTTG	AGAGGCATACAGGGACAACACA	ACACCCCAGCCATGTACGTAGCCATC
	Camk2a	NM_012920	ACCTGCATTTCAGATCACCAGAA	ACACTGTTCCATGACCTCTCACA	ACGCGAGGCCCGCATCTGC
	Cep41	NM_001025770	CTCCGTGCTTGGAACCTTAACT	GCCTCTCGTCGTCATCGTACA	TCACAGAAAAATGCTCATGGCAAGATCATC
	Hif3a	NM_001388512	TGTCCCTGCAGTGACCCTTAC	GGAAGCGATACTGCCCTGTTAC	CCCCTCTCCCCAGTGCTGAGCA
	Hprt1	NM_012583	TGTGCTTGCAGACCAAATACTCTTA	CGTGGATCAAGACGAGACATTG	CCACTGAGTCACCTCCCCAATGCC
	Ift57	NM_001107093	TGTGTGCTTATACCACTAGGATTGG	TCTCCTTTAGAGCAGATTCAATTCC	CACGTTGACCAAATGCACCAGCACA
	Ptk2b	NM_017318	TGACCTTTAACCCGCTCCAT	CGATGTGAGGGTGGTCAAGA	CCTCTCCCCCTCCTTCAGTGATCATGA
	Ywhaz	NM_013011	GGGCACATGTGTCCGATACTG	CACCCTAGGGACAGCTTACAACA	CGCGATTGGATCCCCCGGAAT

Supplementary Table 2. Primer/Probe sequences for ChIP (gDNA) and RNA (hnRNA) analysis. Primer and probe sets were designed using Primer Express 3.0.1 (Life Technologies Corp) and validated. All primer sets generated primer efficiencies of >90%.
