# Supplementary material for: Distinct regulation of hippocampal neuroplasticity and ciliary genes by corticosteroid receptors
Source: Nat Commun. 2021 Aug 6;12:4737. doi: 10.1038/s41467-021-24967-z (PMC8346558; doi:10.1038/s41467-021-24967-z)
Supplement: Supplementary file 2 — Description of Additional Supplementary Files [file 41467_2021_24967_MOESM2_ESM.pdf]

Description of Additional Supplementary Files

File Name: Supplementary Data 1
Description: MR peaks in each sample

File Name: Supplementary Data 2
Description: GR peaks in each sample

File Name: Supplementary Data 3
Description: MR-only MR-GR GR-only peaks

File Name: Supplementary Data 4
Description: MR GR DiffBind Analysis

File Name: Supplementary Data 5
Description: FIMO Analysis

File Name: Supplementary Data 6
Description: RNA-Seq - Intronic Read Counts

File Name: Supplementary Data 7
Description: RNA-Seq - Exonic Read Counts

File Name: Supplementary Data 8
Description: DE Analysis - Intronic Counts

File Name: Supplementary Data 9
Description: DE Analysis - Exonic Counts

File Name: Supplementary Data 10
Description: MR GR vs Intronic Exonic

File Name: Supplementary Data 11
Description: GO Pathway Analysis

File Name: Supplementary Data 12
Description: IPA Analysis
