# Supplementary material for: Distinct regulation of hippocampal neuroplasticity and ciliary genes by corticosteroid receptors
Source: Nat Commun. 2021 Aug 6;12:4737. doi: 10.1038/s41467-021-24967-z (PMC8346558; doi:10.1038/s41467-021-24967-z)
Supplement: Supplementary file 15 — Reporting Summary [file 41467_2021_24967_MOESM15_ESM.pdf]

nature research

Corresponding author(s): Professor Johannes MHM Reul

Last updated by author(s): Jul 7, 2021

Reporting Summary

Nature Research wishes to improve the reproducibility of the work that we publish. This form provides structure for consistency and transparency in reporting. For further information on Nature Research policies, see Authors & Referees and the Editorial Policy Checklist .

Statistics

For all statistical analyses, confirm that the following items are present in the figure legend, table legend, main text, or Methods section.

n/a	Confirmed	
☐	☒	The exact sample size (n) for each experimental group/condition, given as a discrete number and unit of measurement
☐	☒	A statement on whether measurements were taken from distinct samples or whether the same sample was measured repeatedly
☐	☒	The statistical test(s) used AND whether they are one- or two-sided<br>*Only common tests should be described solely by name; describe more complex techniques in the Methods section.*
☒	☐	A description of all covariates tested
☐	☒	A description of any assumptions or corrections, such as tests of normality and adjustment for multiple comparisons
☐	☒	A full description of the statistical parameters including central tendency (e.g. means) or other basic estimates (e.g. regression coefficient) AND variation (e.g. standard deviation) or associated estimates of uncertainty (e.g. confidence intervals)
☐	☒	For null hypothesis testing, the test statistic (e.g. F, t, r) with confidence intervals, effect sizes, degrees of freedom and P value noted<br>*Give P values as exact values whenever suitable.*
☒	☐	For Bayesian analysis, information on the choice of priors and Markov chain Monte Carlo settings
☒	☐	For hierarchical and complex designs, identification of the appropriate level for tests and full reporting of outcomes
☐	☒	Estimates of effect sizes (e.g. Cohen's d, Pearson's r), indicating how they were calculated

Our web collection on statistics for biologists contains articles on many of the points above.

Software and code

Policy information about availability of computer code

Data collection	Not relevant
Data analysis	The ChIP-seq analysis was performed using bcl2fastq (v2.17.1.14), Skewer (version 0.1.125), BWA (version 0.7.15), Bamtools (version 2.3.0), Picard (version 1.111), MACS2 (version 2.1.1.20160309). The RNA-seq analysis was performed using bcl2fastq (v2.17.1.14), Hisat2 (version-2.0.4), featureCounts (implemented in subread-v1.5.0).

For manuscripts utilizing custom algorithms or software that are central to the research but not yet described in published literature, software must be made available to editors/reviewers. We strongly encourage code deposition in a community repository (e.g. GitHub). See the Nature Research guidelines for submitting code & software for further information.

Data

Policy information about availability of data

All manuscripts must include a data availability statement. This statement should provide the following information, where applicable:

- Accession codes, unique identifiers, or web links for publicly available datasets
- A list of figures that have associated raw data
- A description of any restrictions on data availability

The data discussed in this publication have been deposited in NCBI's Gene Expression Omnibus (GEO) and are accessible through GEO Series accession number GSE126706: https://www.ncbi.nlm.nih.gov/geo/query/acc.cgi?acc=GSE126706
The following figures have associated raw data: Figures 1, 2, 4, 5, 6, 7, 8, 9, 10; Supplementary Figures 1, 5, 7, 8, 9, 10. The associated data can be found in the Source Data File.

Field-specific reporting

Please select the one below that is the best fit for your research. If you are not sure, read the appropriate sections before making your selection.

- [x] Life sciences
- [ ] Behavioural & social sciences
- [ ] Ecological, evolutionary & environmental sciences

For a reference copy of the document with all sections, see nature.com/documents/nr-reporting-summary-flat.pdf

Life sciences study design

All studies must disclose on these points even when the disclosure is negative.

Sample size	ChIP-seq studies: n=4; based on experience (Mifsud & Reul, PNAS 2016)<br>RNA-seq studies: n=5-6; based on prior test experiment
Data exclusions	One biological replicate from the BLAM group, RN067, showed a different gene expression profile as compared to other samples in the experiment. We excluded this potential outlier from all downstream analysis as this sample consistently separated from the data and especially the BLAM group in hierarchical clustering as well as principal component analysis (PCA). This statement is also part of our Methods section.
Replication	Our ChIP-seq analyses were conducted using n=4 independent biological samples whereas our Ribo-Zero RNA-seq analyses were done using n=5-6 independent biological samples. Results regarding the genes Fkbp5, Per1 and Sgk1 reflected the MR/GR ChIP and hn/mRNA data published a few years ago by us (Mifsud and Reul (2016) PNAS). In addition, in the manuscript, we present MR and GR ChIP data as well as hnRNA and mRNA data derived from hippocampus tissues collected from a different cohort of rats than the cohort of animals on which our sequencing data are based. These data, presented in Supplementary Figure 3, validate the parallel sequencing data presented in Figure 7.<br>All attempts of replication were successful.
Randomization	Rats and neurospheres were randomly allocated to experimental groups. There were no other experimental subjects.
Blinding	In the Methods, we have indicated blinding for each method and analysis. Blinding was a standard practice except for the animal experiments in which rats were killed under baseline or stress conditions, and in the bioinformatic analysis of the ChIP-seq and RNA-seq data. It should be added that blinding was not relevant for most parts of this study as all ChIP, RNA, plasma and immunocytochemical samples were processed in parallel in exactly the same manner.

Reporting for specific materials, systems and methods

We require information from authors about some types of materials, experimental systems and methods used in many studies. Here, indicate whether each material, system or method listed is relevant to your study. If you are not sure if a list item applies to your research, read the appropriate section before selecting a response.

Materials & experimental systems

n/a	Involved in the study
[ ]	[x] Antibodies
[x]	[ ] Eukaryotic cell lines
[x]	[ ] Palaeontology
[ ]	[x] Animals and other organisms
[ ]	[x] Human research participants
[x]	[ ] Clinical data

Methods

n/a	Involved in the study
[ ]	[x] ChIP-seq
[x]	[ ] Flow cytometry
[x]	[ ] MRI-based neuroimaging

Antibodies

Antibodies used	MR (MR H-300 antibody; sc11412X; Santa Cruz; mix of Lot Nos. C0416, G0616, C2916, K0215) for ChIP-Seq<br>GR (GR H-300 antibody, sc8992X; Santa Cruz; mix of Lot Nos. B2715, K2013, I2515) for ChIP-Seq<br>IgG control (normal rabbit IgG; sc2027X; Santa Cruz); Abcam IgG (ab171870; Lot no. 3353004-1), ProteinTech IgG (30000-0-AP; Lot no. 22000009)); for ChIP<br>MR (rabbit polyclonal; ab64457 (Lot no. GR3207811-1, GR195919-1, GR3323332-1), ab97834 (Lot no. GR3262610-2, GR3262610-10); Abcam); for WB, immunocytochemistry and ChIP<br>GR (rabbit polyclonal; 24050-1-AP (Lot no. 0073937, 00064047); ProteinTech); for WB and ChIP<br>RFX3 (rabbit polyclonal; 14784-1-AP; ProteinTech; Lot no. 00005820); for WB and ChIP<br>Tuj1 (1:500, mouse, Cat. 801202, Biolegend; Lot no. B249869); for immunocytochemistry<br>AC3 (1:400, rabbit, PA535382, ThermoFisher; Lot no. UE2775812); for immunocytochemistry<br>Nestin (1:200, mouse, 4D11, Novus Biologicals; Lot no. 060315); for immunocytochemistry

Validation	The Santa Cruz MR and GR antibodies used for ChIP-seq in this study had been validated by Western blotting and pre-absorption tests followed by ChIP. The results of this validation are published in Mifsud and Reul (2016) PNAS 113:11336-41. This information and the citation are provided in our manuscript.<br>As the polyclonal antibodies of Santa Cruz were discontinued we used MR, GR, IgG, and RFX3 antibodies from Abcam and ProteinTech in the ChIP-qPCR studies reported in our manuscript. Supplementary Figure 8 shows the validation of these antibodies by Western blot analysis. This figure also shows the (negative) results of the ChIPs using non-specific IgGs from Abcam and ProteinTech.

Animals and other organisms

Policy information about studies involving animals; ARRIVE guidelines recommended for reporting animal research

Laboratory animals	Male Wistar rats; 150-175 g on arrival, thus approx. 6 weeks old on arrival; approx. 8 weeks old at the time of experimentation.
Wild animals	Our study did not involve wild animals
Field-collected samples	Not relevant
Ethics oversight	All animal work was approved by the University of Bristol Ethical Committee and the Home Office of the United Kingdom (Animal Scientific Procedures Act, 1986, UK). This is also stated in our manuscript.

Note that full information on the approval of the study protocol must also be provided in the manuscript.

Human research participants

Policy information about studies involving human research participants

Population characteristics	Not relevant
Recruitment	Human fetal cortex tissue was obtained from the SWIFT Human fetal tissue bank at Cardiff University (Cardiff, UK) through medical termination of pregnancy with full donor consent and with ethical approval of the project.
Ethics oversight	Cardiff University Ethics Committee (SWIFT-RTB 46). This information is also provided in the manuscript.

Note that full information on the approval of the study protocol must also be provided in the manuscript.

ChIP-seq

Data deposition

☒ Confirm that both raw and final processed data have been deposited in a public database such as GEO.

☒ Confirm that you have deposited or provided access to graph files (e.g. BED files) for the called peaks.

Data access links<br>*May remain private before publication.*	https://www.ncbi.nlm.nih.gov/geo/query/acc.cgi?acc=GSE126706
Files in database submission	Files in database submission: 360 FASTQ files (with file format "*.fastq.gz"), 24 BED files (with file format ".narrowPeak"), 1 METADATA file (with file format ".xlsx")
Genome browser session<br>(e.g. UCSC)	N/A

Methodology

Replicates	Our ChIP-seq analyses were conducted using n=4 independent biological samples whereas our Ribo-Zero RNA-seq analyses were done using n=5-6 independent biological samples.
Sequencing depth	At least 25 million read pairs were produced per sample (each sample was sequenced on 5 lanes of an Illumina HiSeq4000 machine). At least 24 million read pairs per sample were uniquely mapped. Reads were paired-end and the read length was 75bp.
Antibodies	MR (MR H-300 antibody; sc11412X; Santa Cruz; mix of Lot Nos. C0416, G0616, C2916, K0215)<br>GR (GR H-300 antibody, sc8992X; Santa Cruz; mix of Lot Nos. B2715, K2013, I2515)
Peak calling parameters	Peak calling parameters:<br>MAPPING, STEP 1: "bwa aln -t 8 -q10 rn6_genome sampleID_read1.fastq.gz > sampleID_read1.sai 2> log_sampleID_read1.bwaAln.err"<br>MAPPING, STEP 2: "bwa aln -t 8 -q10 rn6_genome sampleID_read2.fastq.gz > sampleID_read2.sai 2>

log_sampleID_read2.bwaAln.err"
MAPPING, STEP 3: "bwa sampe rn6_genome sampleID_read1.sai sampleID_read2.sai sampleID_read1.fastq.gz sampleID_read2.fastq.gz 2> log.bwaSampe.err | samtools view -bh -o sampleID.bwa.bam -S -"
PEAK CALLING: "macs2 callpeak -t ChIP_file.bam -c Input_file.bam -f BAMPE -g 2.5e9 --verbose 1 --outdir out_folder --name peakset_name"

Data quality

Only peaks with a FDR lower than 5% were reported by the peak caller and used for downstream analyses. The degree of enrichment for the proteins of interest was evaluated through the Normalized Strand Cross-correlation (NSC) and the Relative Strand Cross-correlation (RSC) coefficients, which were calculated for each ChIP sample; in all cases, NSC and RSC values were > 1.05 and > 0.8, respectively, in agreement with the ENCODE guidelines.

Software

The ChIP-seq data was processed using "Skewer" for adapter trimming, "BWA" for mapping, "Bamtools" for read filtering, "Picard" for merging lanes and deduplicating reads, "MACS2" for peak calling.
